# Three phenanthroline–metal complexes with topologically similar but geometrically different conformations

**DOI:** 10.1107/S2056989016016029

**Published:** 2016-10-18

**Authors:** Miguel Angel Harvey, Sebastián Suarez, Ricardo Baggio

**Affiliations:** a Universidad Nacional de la Patagonia S.J.B., Sede Trelew, 9100 Trelew, Chubut, Argentina; b CenPat, CONICET, 9120 Puerto Madryn, Chubut, Argentina; c Departamento de Química Inorgánica, Analítica y Química Física/INQUIMAE-CONICET, Facultad de Ciencias Exactas y Naturales, Universidad de Buenos Aires, Buenos Aires, Argentina; d Departamento de Física, Comisión Nacional de Energía Atómica, Buenos Aires, Argentina

**Keywords:** crystal structure, Cd and Zn complexes, peroxodi­sulfate anion, strong C—H⋯O intra­molecular hydrogen bonds

## Abstract

The structures of three related complexes of general formula *M*(*pds*)(*nab*)_2_ [*pds* is the peroxodi­sulfate anion and *nab* is an nitro­gen-containing aromatic base], *viz*. bis(2,9-dimethyl-1,10-phenanthroline-κ^2^ *N*,*N*′)(peroxodi­sulfato-κ^2^ *O*,*O*′)cadmium, [Cd(S_2_O_8_)(C_14_H_12_N_2_)_2_], (V), bis­(3,4,7,8-tetra­methy-1,10-phenanthroline-κ^2^ *N*,*N*′)(peroxodi­sulfato-κ^2^ *O*,*O*′)zinc, [Zn(S_2_O_8_)(C_16_H_16_N_2_)_2_], (VI), and bis­(3,4,7,8-tetra­methy-1,10-phenanthroline-κ^2^ *N*,*N*′)(peroxodi­sulfato-κ^2^ *O*,*O*′)cadmium, [Cd(S_2_O_8_)(C_16_H_16_N_2_)_2_], (VII), present the same topological coordination, with three chelating ligands in an *M*N_4_O_2_ polyhedron. The main difference resides in the fact that the first two complexes are bis­ected by a crystallographic twofold axis, thus providing a symmetrical environment to the cation, while in the third one this symmetry is disrupted into a clearly unsymmetrical disposition, probably by way of an unusually strong intra­molecular C—H⋯O hydrogen bond. The situation is compared with similar inter­actions in the literature. The structure of (V) is based on a redetermination in the correct space group *C*2/*c* of the structure originally described in the *Cc* space group [Harvey *et al.* (2001). *Aust. J. Chem.* **54**, 307–311; Marsh (2004 ▸). *Acta Cryst.* B**60**, 252–253].

## Abstract 2

Two out of the three very similar complexes described present twofold symmetry but not the third one, probably by way of a strong intra­molecular C—H⋯O hydrogen bond disrupting the symmetry, a fact which is analysed in detail.

## Chemical context   

**Figure 1:**
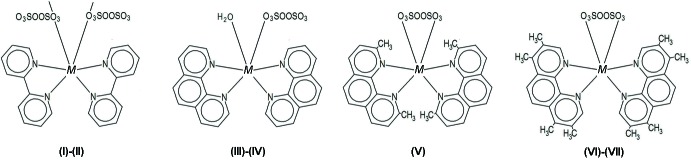
The different coordination modes in the *M*(*pds*)(*nab*)_2_ family. (I): {[Cd(*pds*)(*Bpy*)_2_}_*n*_, *P* 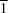 (Harvey *et al.*, 2001*a* ▸); (II): {[Hg(*pds*)(*Bpy*)_2_}_*n*_, *P*2_1_/*n* (Díaz de Vivar *et al.*, 2005 ▸); (III): Cd(*pds*)(*Phen*)_2_(H_2_O) *P* 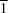 (Harvey *et al.*, 2001*b* ▸); (IV): Zn(*pds*)(*Phen*)_2_(H_2_O), *P* 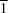 (Harvey *et al.*, 2011 ▸); (V): Cd(*pds*)(*DMPhen*)_2_, *C*2/*c* (Harvey *et al.*, 2001*b* ▸; Marsh, 2004 ▸, and this work); (VI): Zn(*pds*)(*TMPhen*)_2_, *P* 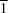 (this work); (VII): Cd(*pds*)(*TMPhen*)_2_, *Pbcn* (this work). Ligand code: *Bpy* = 2,2′-bi­pyridine; *Phen* = 1,10-phenanthroline, *DMPhen* = 2,9-dimethyl-1,10-phenanthroline, *TMPhen* = 3,4,7,8-tetra­methyl-1,10-phenanthroline.

In the last fifteen years we have made several contributions to the structural chemistry of group XII divalent cations, in particular Cd and Zn, complexed by the peroxodi­sulfate anion S_2_O_8_^2−^ (*pds*) and several nitrogen-containing aromatic bases (*nab*). In all these cases, the basic general formula appeared to be *M*(*pds*)(*nab*)_2_, plus the possible inclusion of some water mol­ecules, either coordinating or as a solvate (details of these complexes, including the ones to be described in the present work, are summarized in Fig. 1 ▸). Even if too few structures are reported to make any confident statistical analysis, the results suggest some kind of a trend between the identity of the nitrogen-containing base and the way the *pds* anion performs in coordination. Thus, for the smallest one, *nab* = 2,2′-bi­pyridine (*Bpy*), the structures obtained [(I) and (II)] show two coordinating *pds* units in a bridging –O—S—O– mode. For the inter­mediate *nab* = 1,10-phenanthroline (*Phen*), one of these *pds* appears to be replaced by a (smaller) coord­inating water mol­ecule, while the bound *pds* acts as a pendant monocoordinating ligand [(III) and (IV)]. Finally, at the beginning of this work we had at hand only one single example of a relatively larger *nab* species, represented by *nab* = 2,9-dimethyl-1,10-phenanthroline, [*DMPhen*, (V)], where the single coordinating *pds* folds into itself to bind through both ends, acting in a chelating fashion. Furthermore, in both compounds of each pair of homologues (I)–(II) and (III)–(IV), the anion displays very similar conformations, defined by selected dihedral angles (Harvey *et al.*, 2011 ▸).
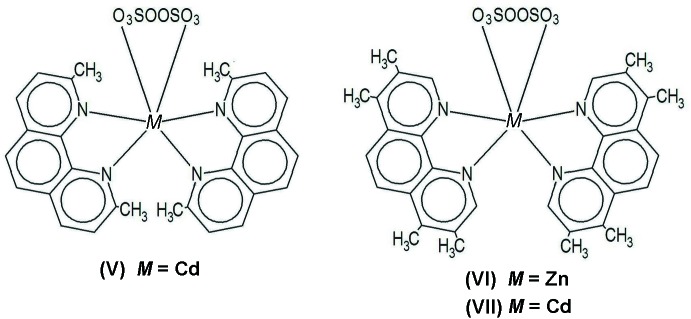


In order to go further in this analysis we synthesized two new complexes of this sort, with *M* = Zn (VI) and *M* = Cd (VII), and with a common, tetra-methyl­ated *nab* ligand, 3,4,7,8-tetra­methyl-1,10-phenanthroline (*TMPhen*). We shall see that they present the same topology as compound (V), but with subtle, inter­esting differences regarding intra­molecular inter­actions which will be discussed in detail. Unlike what happens in the (I)–(II) and (III)–(IV) homologues, in (VI) and (VII) the anion displays remarkably different conformations (Harvey *et al.*, 2011 ▸). Since a comparison with the *DMPhen* structure (V) will be an important part of the discussion, and taking into account that the available data in the correct space group *C*2/*c* [as disclosed by Marsh (2004 ▸)] come from an averaging process (without further refinement) of previous results in *Cc* by our team (Harvey *et al.*, 2001*b* ▸), we indulge in including herein, for completeness, a fresh refinement in *C*2/*c* based on the original data for this structure, in addition to the synthesis and crystal structure of the two new complexes, (VI) and (VII). Even though we shall restrict this discussion to the *pds* anion, it is pertinent to state that the tetra­thio­nate anion (S_4_O_6_^2−^) behaves in a rather similar way, and that the tetra­thio­nate Zn and Cd complexes with *nab* = *DMBpy* = 4,4′-dimethyl-2,2′-bi­pyridine (Harvey *et al.*, 2013 ▸) have a similar coordination disposition to (V), (VI) and (VII).

## Structural commentary   

The Zn complex (VI) crystallizes in space group *Pbca*, and the complete molecule is bis­ected by a twofold symmetry axis, hence only half of the mol­ecule is independent (*Z*′ = 1/2); even if in a different space group, these properties are shared by structure (V). The Cd counterpart (VII), in turn, crystallizes in space group *P* 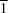 with a full mol­ecule in the asymmetric unit.

**Figure 2:**
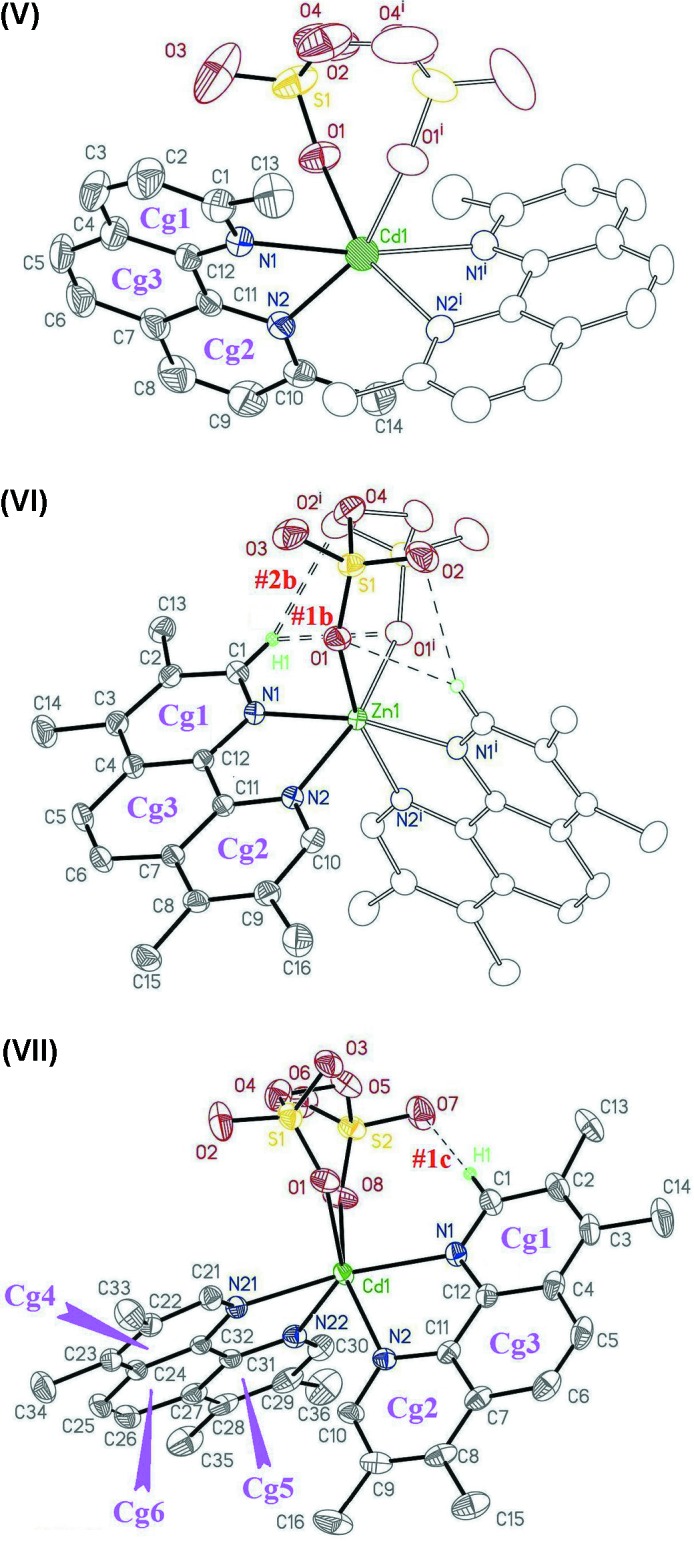
Ellipsoid plots of (V), (VI) and (VII), drawn at the 50% probability level. Only the H atoms involved in intra­molecular hydrogen bonds (dashed lines) are shown. Symmetry code for (V) and (VI): (i) −*x*, *y*, 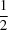 − *z*.

All three compounds present a topologically similar mol­ecular configuration (Fig. 2 ▸), consisting in a central cation to which three bidentate chelating ligands bind, *viz*. two *N,N′-nab* and one *O,O′-pds* units. In particular, the ‘close’ character of the *pds* anion is in line with the trend so far observed, that methyl­ated bases favour the chelating behaviour of *pds*.

Coordination distances in all three compounds are basically featureless, and agree with the expected values for each cation–ligand pair. However, a difference arises in the asymmetric way in which the ligands bind in (VII), contrasting dramatically with the twofold arrangement in (V) and (VI).

The chelating character of the ligands involved induces highly distorted coordination polyhedra. Proof of this is presented in Table 1 ▸, which shows the departure of the ‘*trans’* angles in (V), (VI) and (VII) from their expected values of 180° for a regular octa­hedron. This makes the polyhedra difficult to classify, and impairs the description of coordination in terms of any regular model. In this regard, all three compounds are suitable for the analysis *via* the Vectorial Bond Valence Model (VBVM) suggested by Harvey *et al.* (2006 ▸), an approach tending to a simpler description of multidentate binding, in which the action of each ligand is integrated into a single inter­action vector, or VBV (Vectorial Bond Valence), derived from the individual bond valences of the coordinating atoms. VBVM predicts a nil resultant of the vectorial sum of all the VBV vectors and, as a consequence, in this particular case of three-ligand coordination geometry, their disposition in a planar array. The first condition is complied satisfactorily with a very short resultant for the Bond Valence Vectors [0.08, 0.03 and 0.08 valence units for (V), (VI) and (VII), respectively]. The second requirement (planar array of vectors), applies *sensu stricto* in (V) and (VI), due to the intrinsic twofold symmetry around the cation, and it falls well within experimental error in (VII), where the calculated angles between Bond Valence Vectors add up to 359.5 (3)° and the plane defined by their extremes leave the Cd^II^ atom only 0.09 (2) Å aside.

**Figure 3:**
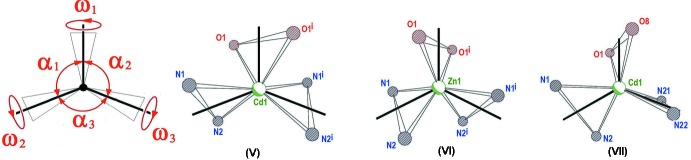
Schematic representation of the ligand distortion. In open bonds, the chelating ligands, (drawn as connected to each other, for clarity); in solid lines, the VBV vectors, representing the integrated action of each ligand. Angle codes are explained in the text.

As an unwitting bonus of this description, these planes appear as a natural reference frame for describing ligand orientations in the polyhedra, evidencing in (V) and (VI) their adherence to twofold symmetry and in (VII) significant departures from a symmetric arrangement. This can be visualized in Fig. 3 ▸, where a schematic representation (with an exaggerated perspective) is made of the ligand bites (open bonds) as well as the VBV representing their joint effect as a ligand (solid lines). At the left, the explanation of a group of angles helping to describe the orientation of the coordination planes is provided: angles labeled α give account of the angular separation in the plane between vectors, while those labeled ω measure the out-of-plane rotation of the coordination planes around the corresponding VBV vectors. It is apparent, either by visual inspection of Fig. 3 ▸ or through the analysis of the ω values (Table 2 ▸), that the coordination polyhedron in (VII) is abnormally distorted. Since this could be the result of packing strain (inter­molecular inter­actions) or just due to genuine intra­molecular forces, we shall analyze and compare the three packing arrangements for (V), (VI) and (VII).

## Supra­molecular features   

The most relevant, non-covalent inter­actions involved are presented in Table 3 ▸ (hydrogen bonds) and Table 4 ▸ (π–π contacts). The second column includes a code, which labels each inter­action for easy reference; in the last column, the role the inter­action plays in packing is listed.

**Figure 4:**
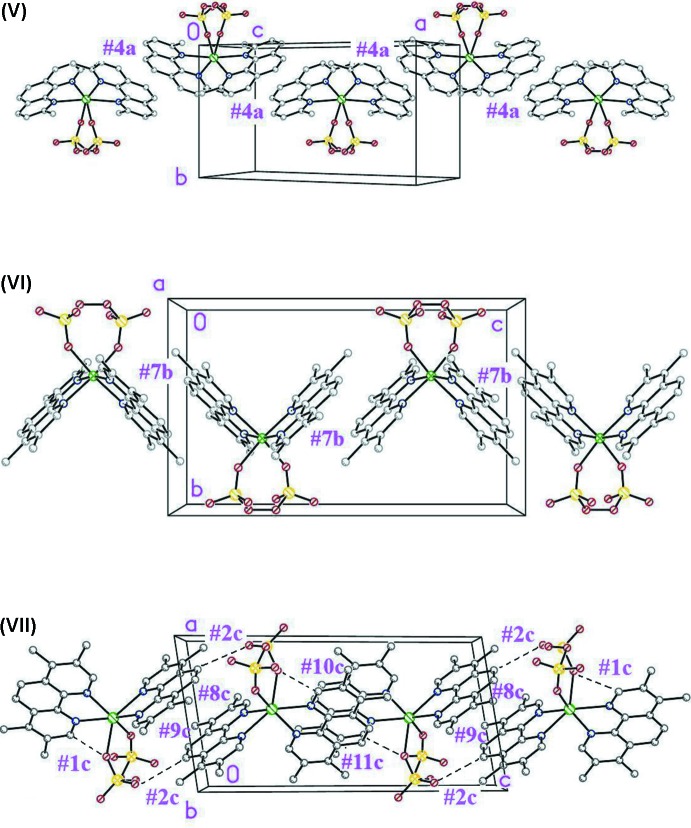
The π-bonded one-dimensional leitmotifs in all three structures. Stacking inter­actions labeled as in Table 4 ▸. H atoms have been omitted for clarity.

Fig. 4 ▸ presents packing views of all three structures: it is apparent that in spite of crystallizing in different space groups, with different symmetry environments, the *leitmotifs* are strictly the same, *viz.* π–π bound chains running along [10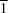] in (V) and [001] in (VI) and (VII), the link being the stacking inter­action appearing in Table 4 ▸, which in all cases connect inversion-related moieties. Except for the rather strong #2*c* in (VII), the remaining inter­molecular inter­actions are weak and serve either to strengthen the link within the chains (marked as ‘intra­chain’ in the tables) or to weakly connect parallel chains with each other (‘inter­chain’) to end up defining weakly bound three-dimensional structures. This description is valid for all three structures, and there is nothing special about the packing inter­actions in (VII) so as to ascribe to them the responsibility for the coordination ‘anomaly’. In fact, inter­action #2*c*, which due to its outstanding character might be thought of as a candidate to blame, involves the ‘well behaved’ *N21,N22-TMPhen* and not the one departing from geom­etrical regularity (*N1,N2-TMPhen*). This fact can be clearly appreciated in Fig. 4 ▸ (bottom).

As far as intra­molecular inter­actions are concerned, the symmetric cases (V) and (VI) present different behaviours regarding these contacts. Methyl groups at the 2,9 positions inhibit structure (V) from entering into any significant (C—H)_arom_⋯O_pds_ intra­molecular contact, as suggested in Fig. 2 ▸ and disclosed in Table 3 ▸, where only weak, inter­molecular inter­actions are to be found. Structure (VI), in turn, having sites 2 and 9 free, is amenable of a closer approach of (C—H)_arom_ donors and O_pds_ acceptors, and in fact a pair of weak bonds set up (#1*b* and #2*b*, Fig. 2 ▸ and Table 3 ▸). However, it is in structure (VII) where things depart from normal, with a second unusually short and almost straight C—H⋯O bond inter­nally linking the ‘offending’ *N1,N2-TMPhen* ligand and the *pds* anion in the same coordination sphere (inter­action #1*c* in Table 3 ▸). In order to evaluate, at least in comparative terms the real significance of this bond (and, by extension, the similar #2*c*), we made some CSD (Version 5.37; Groom *et al.*, 2016 ▸) data mining and statistical comparisons.

**Figure 5:**
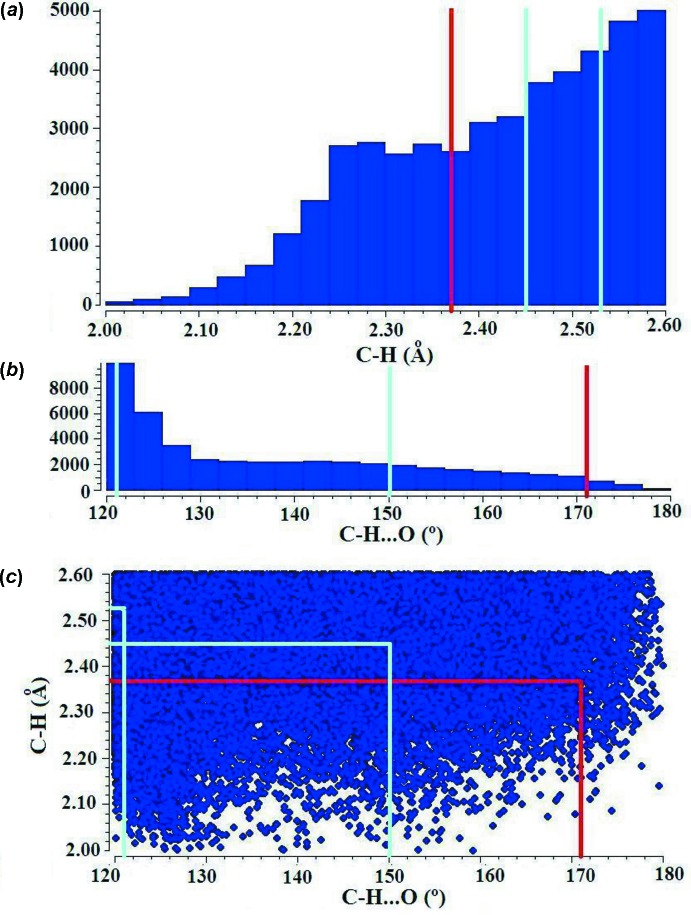
Statistical analysis of intra­molecular (C—H)_arom_⋯O bonds as found in the literature. In cyan, those found in (VI); in red, the one in (VII).

When comparing inter­action #1*c* with its peers in the database, we looked for (C—H)_arom_⋯O intra­molecular bonds with almost no restrictions (*viz.* 2 Å < H⋯O < 3.0 Å; 120° < C—H⋯O < 180°). The results (from *ca* 30000 entries analysed) are quoted in Fig. 5 ▸, where the distance (*a*) and angle (*b*) histograms, as well as the combined scatterplot (*c*) are presented. The two hydrogen bonds in (VI), marked in cyan, appear to be absolutely average, as are their structural consequences. The one in (VII) (marked in red), instead appears endowed with a rather unique character, in particular its nearly straight C—H⋯O configuration. We tried to evaluate how frequent this kind of disrupting behaviour was (in terms of mol­ecular distortions) among comparable C—H⋯O inter­actions. Inspection of the occurrences found showed that they tended to appear either in monocoordinating ligands or pendant groups, in all cases with free rotations at some point in the chain, which made the C—H⋯O contact almost irrelevant in terms of configurational energy. What makes the case in (VII) unusual is the chelating character of the ligands involved, with the concomitant deformation of the coordination polyhedron.

Summarizing, there are in principle two possible reasons for the mol­ecular geometry in (VII): either the (packing-assisted) asymmetry with which ligand *(N1,N2)TMPhen* binds Cd1 is the reason allowing for an unusual closeness between C1—H1 and O7, giving room to a strong hydrogen bond, or (the other way round) it is this hydrogen bond that is the cause, and the asymmetric coordination its concomitant consequence. The lack of significant inter­molecular packing inter­actions which may justify the distortion in (VII), in addition to the outstanding character of the #1*c* C—H⋯O bond seem to sustain the latter hypothesis, *viz.* that it is the presence of this hydrogen bond (‘weak’ among ‘strong’ but ‘strong’ among ‘weak’) which disrupts the expected symmetrical geometry in the Cd(*pds*)(*TMPhen*)_2_ unit, constituting thus a rare case of a non-conventional C—H⋯O bond being responsible for a surprising mol­ecular configuration.

## Synthesis and crystallization   

Compounds (VI) and (VII) were synthesized in a similar fashion: a solution (4 ml) containing 0.050 mmol (13.5 mg) of potassium peroxodi­sulfate and 0.100 mmol (23.6 mg) of 3,4,7,8-tetra­methyl-1,10- phenanthroline (in a 3:1 *v*/*v* methanol:water mixture) were added to 0.050 mmol of the corres­ponding metal acetate [Zn(OAc)_2_: 11.0 mg; Cd(OAc)_2_: 13.3 mg). An initial precipitate of extremely small needles was readily digested, but in a few days a crop of single crystals suitable for X-ray diffraction were obtained, in the form of colorless blocks. For the synthesis of (V), see Harvey *et al.* (2001*b* ▸).

## Refinement details   

Data collection details and refinement results for (V), (VI) and (VII) are summarized in Table 5 ▸. The data set for (V) is the same used in the original publication (Harvey *et al.*, 2001*b* ▸) reporting the structure refined in the *C*c space group. All hydrogen atoms were found in a difference Fourier map, but further idealized and allowed to ride on their parent atoms with C—H = 0.93–0.98 Å, and *U*_iso_(H) = 1.2*U*_eq_(C) or 1.5*U*_eq_(C) for methyl H atoms. A rotating model was used for the methyl groups. For (V), a soft restraint in displacement factors was applied (RIGU in *SHELXL2014*).

## Supplementary Material

Crystal structure: contains datablock(s) V, VI, VII, global. DOI: 10.1107/S2056989016016029/rz5195sup1.cif

Structure factors: contains datablock(s) V. DOI: 10.1107/S2056989016016029/rz5195Vsup2.hkl

Structure factors: contains datablock(s) VI. DOI: 10.1107/S2056989016016029/rz5195VIsup3.hkl

Structure factors: contains datablock(s) VII. DOI: 10.1107/S2056989016016029/rz5195VIIsup4.hkl

CCDC references: 1509297, 1509298, 1509299

Additional supporting information:  crystallographic information; 3D view; checkCIF report

## Figures and Tables

**Table 1:** Selected geometric parameters (Å, °) for (V), (VI) and (VII)

(V)			
Cd1—N2	2.307 (2)	N1—Cd1—N1^i^	171.15 (10)
Cd1—O1	2.314 (2)	O1—Cd1—N2^i^	161.06 (8)
Cd1—N1	2.409 (3)	O1^i^—Cd1—N2	161.06 (8)
			
(VI)			
Zn1—N1	2.0950 (14)	N1—Zn1—N1^i^	168.3 (2)
Zn1—O1	2.1476 (13)	N2—Zn1—O1^i^	167.8 (2)
Zn1—N2	2.1853 (14)	N2^i^—Zn1—O1	167.8 (2)
			
(VII)			
Cd1—N1	2.3075 (19)	O8—Cd1—N2	158.5 (2)
Cd1—N2	2.3278 (19)	N1—Cd1—N21	152.0 (2)
Cd1—O8	2.3232 (18)	N22–Cd1—O1	142.4 (2)
Cd1—N22	2.3304 (19)		
Cd1—N21	2.327 (2)		
Cd1—O1	2.3371 (19)		

Symmetry code for (V) and (VI): (i) −*x*, *y*, −*z* + 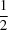.

**Table 2:** Distortion angles as defined in Fig. 3 ▸ (°)

Angle	(V)	(VI)	(VII)
α1	111.1	116.7	119.0
α2	111.1	116.7	111.0
α3	137.9	126.6	129.4
ω1	49.0	67.5	71.1
ω2	55.9	55.8	27.0
ω3	55.9	55.8	84.7

**Table 3:** Hydrogen-bond geometry (Å, °) for (V), (VI) and (VII) *Cg*1, *Cg*3, *Cg*4 and *Cg*6 are the centroids of the N1/C1–C4/C12, C4–C7/C11/C12, N21/C21–C24/C32 and C24–C27/C31/C32 rings, respectively.

Structure	Label	*D*—H⋯*A*	*D*—H	H⋯*A*	*D*⋯*A*	*D*—H⋯*A*	Character
(V)							
	#1*a*	C3—H3⋯O3^ii^	0.93	2.54	3.228 (5)	131	Inter­chain
	#2*a*	C14—H14*B*⋯O2^iii^	0.96	2.45	3.397 (5)	167	Inter­chain
	#3*a*	C14—H14*C*⋯O2^iv^	0.96	2.54	3.331 (5)	140	Inter­chain
	#4*a*	C13—H13⋯O1	0.96	2.71	3.099 (2)	105	Intra­molecular
	#5*a*	C13—H13⋯O2	0.96	2.78	3.667 (2)	155	Intra­molecular
(VI)							
	#1*b*	C1—H1⋯O1^i^	0.93	2.53	3.117 (2)	121	Intra­molecular
	#2*b*	C1—H1⋯O2^i^	0.93	2.45	3.286 (2)	150	Intra­molecular
	#3*b*	C15—H15*B*⋯O3^ii^	0.96	2.51	3.451 (2)	166	Inter­chain
	#4*b*	C13—H13*B*⋯O2^iii^	0.96	2.59	3.324 (2)	133	Inter­chain
	#5*b*	C16—H16*B*⋯O2^iv^	0.96	2.59	3.543 (3)	172	Inter­chain
	#6*b*	C13—H13*A*⋯*Cg*3^v^	0.96	2.73	3.9857	127	Inter­chain
(VII)							
	#1*c*	C1—H1⋯O7	0.93	2.37	3.296 (3)	171	Intra­molecular
	#2*c*	C26—H26⋯O2^i^	0.93	2.29	3.204 (3)	165	Intra­chain
	#3*c*	C15—H15*B*⋯*Cg*1^iv^	0.96	2.89	3.578 (4)	129	Intra­chain
	#4*c*	C34—H34*C*⋯*Cg*4^i^	0.96	2.88	3.599 (3)	133	Intra­chain
	#5*c*	C36—H36*B*⋯O6^ii^	0.96	2.47	3.165 (4)	130	Inter­chain
	#6*c*	C13—H13*A*⋯*Cg*1^iii^	0.96	2.93	3.607 (4)	128	Inter­chain
	#7*c*	C35—H35*C*⋯*Cg*6^v^	0.96	2.69	3.604 (4)	158	Inter­chain

Symmetry codes for (V): (ii) 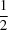 − *x*, −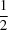 − *y*, 1 − *z*; (iii) −*x*, −*y*, −*z*; (iv) *x*, 1 + *y*, *z*. Symmetry codes for (VI): (i) −*x*, *y*, −*z* + 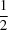; (ii) *x*, *y* − 1, *z*; (iii) *x* + 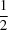, *y* − 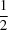, −*z* + 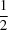; (iv) −*x* − 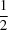, *y* − 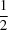, *z*; (v) −*x* + 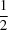, *y* + 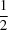, *z*. Symmetry codes for (VII): (i) −*x* + 1, −*y* + 1, −*z* + 2; (ii) *x* + 1, *y*, *z*; (iii) −*x* + 1, −*y*, −*z* + 1; (iv) −*x* + 1, −*y* + 1, −*z* + 1; (v) −*x* + 2, −*y* + 1, −*z* + 2.

**Table 4:** π–π contacts (Å, °) for (V), (VI) and (VII) ccd: centroid-to-centroid distance; da: dihedral angle between planes, sa: slippage angle (average angle subtended by the inter­centroid vector to the plane normal), ipd: inter­planar distance (average distance from one plane to the neighbouring centroid); for details, see Janiak (2000 ▸). *Cg*1, *Cg*2, *Cg*3, *Cg*4 and *Cg*6 are the centroids of the N1/C1–C4/C12, N2/C7–C11, C4–C7/C11/C12, N21/C21–C24/C32 and C24–C27/C31/C32 rings, respectively.

Structure	Label	*Cg*⋯*Cg*	ccd	da	sa	ipd	Character
(V)							
	#6a	*Cg*1⋯*Cg*3^v^	3.823 (3)	0.95 (14)	15.0(1.6)	3.69 (3)	Intra­chain
(VI)							
	#7b	*Cg*2⋯*Cg*3^vi^	3.8101 (10)	2.34 (8)	25.5 (7)	3.44 (2)	Intra­chain
(VII)							
	#8c	*Cg*2⋯*Cg*3^v^	3.737 (3)	0.9 (2)	21.3 (7)	3.48 (2)	Intra­chain
	#9c	*Cg*3⋯*Cg*3^v^	3.717 (3)	0	21.5	3.4577 (9)	Intra­chain
	#10*c*	*Cg*4⋯*Cg*6^vi^	3.700 (2)	0.6 (2)	21.8 (3)	3.43 (2)	Intra­chain
	#11*c*	*Cg*6⋯*Cg*6^vi^	3.669 (2)	0	20.9	3.4269 (9)	Intra­chain

Symmetry code for (V): (v) 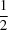 − *x*, 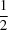 − *y*, 1 − *z*. Symmetry code for (VI): (vi) −*x*, −*y*, −*z*. Symmetry codes for (VII): (v) 1 − *x*, 1 − *y*, 1 − *z*; (vi) 1 − *x*, 1 − *y*, 2 − *z*.

**Table 5:** Experimental details

	(V)	(VI)	(VII)
Crystal data			
Chemical formula	[Cd(S_2_O_8_)(C_14_H_12_N_2_)]	[Zn(S_2_O_8_)(C_16_H_16_N_2_)_2_]	[Cd(S_2_O_8_)(C_16_H_16_N_2_)_2_]
*M*_r_	721.03	730.10	777.13
Crystal system, space group	Monoclinic, *C*2/*c*	Orthorhombic, *P* *b* *c* *n*	Triclinic, *P* 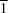
Temperature (K)	296	294	294
*a*, *b*, *c* (Å)	22.233 (12), 9.566 (5), 16.017 (8)	15.6244 (2), 10.8803 (2), 17.9446 (3)	8.601 (3), 11.063 (4), 16.932 (5)
α, β, γ (°)	90, 123.78 (3), 90	90, 90, 90	98.788 (5), 97.713 (5), 97.943 (5)
*V* (Å^3^)	2831 (3)	3050.55 (9)	1557.0 (9)
*Z*	4	4	2
Radiation type	Mo *K*α	Mo *K*α	Mo *K*α
μ (mm^−1^)	0.98	1.00	0.90
Crystal size (mm)	0.80 × 0.30 × 0.15	0.35 × 0.20 × 0.16	0.28 × 0.16 × 0.14

Data collection			
Diffractometer	Siemens R3m	Oxford Diffraction Gemini CCD S Ultra	Oxford Diffraction Gemini CCD S Ultra
Absorption correction	ψ scan (*P3/P4-PC*; Siemens, 1991 ▸)	Multi-scan (*CrysAlis PRO*; Oxford Diffraction, 2009 ▸)	Multi-scan (*CrysAlis PRO*; Oxford Diffraction, 2009 ▸)
*T*_min_, *T*_max_	0.70, 0.88	0.76, 0.84	0.76, 0.84
No. of measured, independent and observed [*I* > 2σ(*I*)] reflections	2562, 2495, 2300	63344, 3988, 3341	41025, 7888, 6692
*R*_int_	0.040	0.049	0.057
(sin θ/λ)_max_ (Å^−1^)	0.595	0.688	0.696

Refinement			
*R*[*F* ^2^ > 2σ(*F* ^2^)], *wR*(*F* ^2^), *S*	0.027, 0.073, 1.11	0.033, 0.092, 1.04	0.033, 0.071, 1.07
No. of reflections	2495	3988	7888
No. of parameters	197	217	432
No. of restraints	195	0	0
H-atom treatment	H-atom parameters constrained	H-atom parameters constrained	H-atom parameters constrained
Δρ_max_, Δρ_min_ (e Å^−3^)	0.47, −0.41	0.47, −0.53	0.54, −0.57

Computer programs: *P3/P4-PC* (Siemens, 1991 ▸), *CrysAlis PRO* (Oxford Diffraction, 2009 ▸), *SHELXS97* and *SHELXTL* (Sheldrick, 2008 ▸), *SHELXL2014* (Sheldrick, 2015 ▸) and *PLATON* (Spek, 2009 ▸).

## supplementary crystallographic information

### (V) Bis(2,9-dimethyl-1,10-phenanthroline-κ2N,N')(peroxodisulfato-κ2O,O')cadmium(II) Crystal data

**Table:**

[Cd(S_2_O_8_)(C_14_H_12_N_2_)]	*F*(000) = 1456
*M**_r_* = 721.03	*D*_x_ = 1.691 Mg m^−^^3^
Monoclinic, *C*2/*c*	Mo *K*α radiation, λ = 0.71073 Å
*a* = 22.233 (12) Å	Cell parameters from 40 reflections
*b* = 9.566 (5) Å	θ = 7.5–15°
*c* = 16.017 (8) Å	µ = 0.98 mm^−^^1^
β = 123.78 (3)°	*T* = 296 K
*V* = 2831 (3) Å^3^	Block, colourless
*Z* = 4	0.80 × 0.30 × 0.15 mm

### (V) Bis(2,9-dimethyl-1,10-phenanthroline-κ2N,N')(peroxodisulfato-κ2O,O')cadmium(II) Data collection

**Table:**

Siemens R3m diffractometer	2300 reflections with *I* > 2σ(*I*)
Radiation source: fine-focus sealed tube	*R*_int_ = 0.040
Graphite monochromator	θ_max_ = 25.0°, θ_min_ = 2.2°
ω/2θ scans	*h* = 0→26
Absorption correction: ψ scan (*P3/P4-PC*; Siemens, 1991)	*k* = 0→11
*T*_min_ = 0.70, *T*_max_ = 0.88	*l* = −19→15
2562 measured reflections	2 standard reflections every 98 reflections
2495 independent reflections	intensity decay: 2%

### (V) Bis(2,9-dimethyl-1,10-phenanthroline-κ2N,N')(peroxodisulfato-κ2O,O')cadmium(II) Refinement

**Table:**

Refinement on *F*^2^	195 restraints
Least-squares matrix: full	Hydrogen site location: inferred from neighbouring sites
*R*[*F*^2^ > 2σ(*F*^2^)] = 0.027	H-atom parameters constrained
*wR*(*F*^2^) = 0.073	*w* = 1/[σ^2^(*F*_o_^2^) + (0.0424*P*)^2^ + 2.2422*P*] where *P* = (*F*_o_^2^ + 2*F*_c_^2^)/3
*S* = 1.11	(Δ/σ)_max_ < 0.001
2495 reflections	Δρ_max_ = 0.47 e Å^−^^3^
197 parameters	Δρ_min_ = −0.41 e Å^−^^3^

### (V) Bis(2,9-dimethyl-1,10-phenanthroline-κ2N,N')(peroxodisulfato-κ2O,O')cadmium(II) Special details

**Table:**

Geometry. All esds (except the esd in the dihedral angle between two l.s. planes) are estimated using the full covariance matrix. The cell esds are taken into account individually in the estimation of esds in distances, angles and torsion angles; correlations between esds in cell parameters are only used when they are defined by crystal symmetry. An approximate (isotropic) treatment of cell esds is used for estimating esds involving l.s. planes.

### (V) Bis(2,9-dimethyl-1,10-phenanthroline-κ2N,N')(peroxodisulfato-κ2O,O')cadmium(II) Fractional atomic coordinates and isotropic or equivalent isotropic displacement parameters (Å^2^)

**Table:**

	*x*	*y*	*z*	*U*_iso_*/*U*_eq_	
Cd	0.0000	0.08973 (3)	0.2500	0.02566 (11)	
S1	0.07113 (5)	−0.23114 (8)	0.22102 (7)	0.0491 (2)	
O1	0.04563 (12)	−0.0900 (2)	0.20412 (17)	0.0433 (5)	
O2	0.04309 (19)	−0.3067 (3)	0.1303 (2)	0.0819 (9)	
O3	0.14664 (16)	−0.2448 (4)	0.2948 (2)	0.0944 (10)	
O4	0.04040 (15)	−0.3065 (3)	0.2828 (2)	0.0699 (8)	
N1	0.11739 (11)	0.0703 (2)	0.40497 (16)	0.0289 (4)	
N2	0.08013 (11)	0.2280 (2)	0.23846 (16)	0.0278 (4)	
C1	0.13651 (16)	0.0027 (3)	0.4888 (2)	0.0378 (6)	
C2	0.20758 (18)	−0.0459 (4)	0.5553 (2)	0.0518 (8)	
H2	0.2192	−0.0960	0.6122	0.062*	
C3	0.25910 (17)	−0.0202 (4)	0.5371 (2)	0.0504 (7)	
H3	0.3057	−0.0549	0.5801	0.060*	
C4	0.24159 (15)	0.0594 (3)	0.4528 (2)	0.0376 (6)	
C5	0.29429 (16)	0.0998 (3)	0.4334 (3)	0.0451 (7)	
H5	0.3417	0.0686	0.4758	0.054*	
C6	0.27702 (15)	0.1821 (3)	0.3550 (2)	0.0460 (7)	
H6	0.3125	0.2076	0.3443	0.055*	
C7	0.20481 (14)	0.2306 (3)	0.2885 (2)	0.0360 (6)	
C8	0.18446 (17)	0.3241 (3)	0.2091 (3)	0.0473 (7)	
H8	0.2188	0.3552	0.1974	0.057*	
C9	0.11524 (18)	0.3689 (3)	0.1499 (3)	0.0479 (7)	
H9	0.1025	0.4334	0.0991	0.057*	
C10	0.06267 (15)	0.3185 (3)	0.1647 (2)	0.0344 (6)	
C11	0.15031 (13)	0.1874 (3)	0.30242 (19)	0.0267 (5)	
C12	0.16911 (14)	0.1021 (3)	0.3876 (2)	0.0282 (5)	
C13	0.08122 (18)	−0.0186 (4)	0.5129 (2)	0.0529 (8)	
H13A	0.0432	0.0490	0.4773	0.079*	
H13B	0.1035	−0.0075	0.5838	0.079*	
H13C	0.0614	−0.1110	0.4931	0.079*	
C14	−0.01427 (16)	0.3639 (3)	0.0986 (2)	0.0431 (7)	
H14A	−0.0436	0.3085	0.1126	0.065*	
H14B	−0.0306	0.3519	0.0295	0.065*	
H14C	−0.0181	0.4607	0.1109	0.065*	

### (V) Bis(2,9-dimethyl-1,10-phenanthroline-κ2N,N')(peroxodisulfato-κ2O,O')cadmium(II) Atomic displacement parameters (Å^2^)

**Table:**

	*U*^11^	*U*^22^	*U*^33^	*U*^12^	*U*^13^	*U*^23^
Cd	0.02103 (15)	0.02505 (15)	0.03202 (16)	0.000	0.01544 (12)	0.000
S1	0.0680 (5)	0.0367 (4)	0.0609 (5)	0.0191 (4)	0.0471 (4)	0.0096 (3)
O1	0.0568 (13)	0.0337 (10)	0.0519 (12)	0.0094 (8)	0.0380 (11)	0.0020 (8)
O2	0.139 (3)	0.0502 (14)	0.0877 (16)	−0.0057 (16)	0.0822 (17)	−0.0174 (13)
O3	0.0681 (14)	0.120 (3)	0.0945 (19)	0.0418 (15)	0.0448 (13)	0.0377 (18)
O4	0.0990 (19)	0.0534 (14)	0.0872 (19)	0.0259 (13)	0.0703 (17)	0.0269 (13)
N1	0.0253 (9)	0.0300 (11)	0.0295 (9)	0.0008 (8)	0.0141 (8)	0.0000 (8)
N2	0.0260 (9)	0.0255 (10)	0.0333 (10)	−0.0022 (7)	0.0174 (8)	−0.0011 (8)
C1	0.0420 (13)	0.0373 (14)	0.0302 (11)	−0.0003 (10)	0.0176 (10)	0.0023 (10)
C2	0.0444 (13)	0.0607 (19)	0.0385 (15)	0.0076 (12)	0.0158 (11)	0.0121 (14)
C3	0.0363 (14)	0.0574 (18)	0.0426 (14)	0.0092 (13)	0.0127 (12)	0.0083 (13)
C4	0.0254 (11)	0.0390 (14)	0.0390 (12)	0.0040 (9)	0.0121 (9)	−0.0051 (10)
C5	0.0246 (12)	0.0519 (17)	0.0529 (15)	0.0008 (11)	0.0180 (12)	−0.0072 (12)
C6	0.0285 (12)	0.0529 (16)	0.0584 (15)	−0.0030 (11)	0.0252 (11)	−0.0071 (12)
C7	0.0308 (11)	0.0348 (13)	0.0496 (13)	−0.0071 (9)	0.0270 (10)	−0.0071 (11)
C8	0.0467 (14)	0.0466 (16)	0.0616 (16)	−0.0057 (12)	0.0382 (13)	0.0037 (13)
C9	0.0499 (13)	0.0442 (16)	0.0602 (18)	−0.0026 (11)	0.0372 (13)	0.0136 (14)
C10	0.0387 (12)	0.0275 (12)	0.0384 (12)	−0.0024 (9)	0.0224 (10)	0.0036 (10)
C11	0.0241 (10)	0.0243 (11)	0.0324 (11)	−0.0032 (8)	0.0161 (8)	−0.0065 (9)
C12	0.0252 (10)	0.0259 (11)	0.0321 (11)	−0.0014 (8)	0.0150 (9)	−0.0056 (9)
C13	0.0528 (17)	0.069 (2)	0.0405 (17)	−0.0018 (16)	0.0284 (15)	0.0095 (16)
C14	0.0409 (14)	0.0390 (15)	0.0449 (16)	0.0026 (11)	0.0211 (12)	0.0121 (13)

### (V) Bis(2,9-dimethyl-1,10-phenanthroline-κ2N,N')(peroxodisulfato-κ2O,O')cadmium(II) Geometric parameters (Å, º)

**Table:**

Cd—N2	2.307 (2)	C4—C12	1.408 (4)
Cd—N2^i^	2.308 (2)	C4—C5	1.422 (4)
Cd—O1^i^	2.314 (2)	C5—C6	1.344 (5)
Cd—O1	2.314 (2)	C5—H5	0.9300
Cd—N1	2.409 (3)	C6—C7	1.423 (4)
Cd—N1^i^	2.409 (3)	C6—H6	0.9300
S1—O2	1.419 (3)	C7—C8	1.407 (4)
S1—O3	1.423 (3)	C7—C11	1.411 (4)
S1—O1	1.431 (2)	C8—C9	1.352 (5)
S1—O4	1.648 (3)	C8—H8	0.9300
O4—O4^i^	1.494 (6)	C9—C10	1.402 (4)
N1—C1	1.329 (3)	C9—H9	0.9300
N1—C12	1.359 (4)	C10—C14	1.491 (4)
N2—C10	1.336 (3)	C11—C12	1.438 (4)
N2—C11	1.363 (3)	C13—H13A	0.9600
C1—C2	1.405 (4)	C13—H13B	0.9600
C1—C13	1.494 (4)	C13—H13C	0.9600
C2—C3	1.352 (5)	C14—H14A	0.9600
C2—H2	0.9300	C14—H14B	0.9600
C3—C4	1.404 (4)	C14—H14C	0.9600
C3—H3	0.9300		
			
N2—Cd—N2^i^	110.03 (11)	C3—C4—C5	122.4 (3)
N2—Cd—O1^i^	161.06 (8)	C12—C4—C5	120.1 (3)
N2^i^—Cd—O1^i^	84.62 (8)	C6—C5—C4	121.4 (3)
N2—Cd—O1	84.62 (8)	C6—C5—H5	119.3
N2^i^—Cd—O1	161.06 (8)	C4—C5—H5	119.3
O1^i^—Cd—O1	84.03 (11)	C5—C6—C7	120.5 (3)
N2—Cd—N1	71.60 (8)	C5—C6—H6	119.7
N2^i^—Cd—N1	113.83 (8)	C7—C6—H6	119.7
O1^i^—Cd—N1	91.75 (8)	C8—C7—C11	117.1 (3)
O1—Cd—N1	81.65 (8)	C8—C7—C6	123.1 (3)
N2—Cd—N1^i^	113.83 (8)	C11—C7—C6	119.7 (3)
N2^i^—Cd—N1^i^	71.61 (8)	C9—C8—C7	120.3 (3)
O1^i^—Cd—N1^i^	81.65 (8)	C9—C8—H8	119.8
O1—Cd—N1^i^	91.75 (8)	C7—C8—H8	119.8
N1—Cd—N1^i^	171.15 (10)	C8—C9—C10	120.2 (3)
O2—S1—O3	116.6 (2)	C8—C9—H9	119.9
O2—S1—O1	112.62 (17)	C10—C9—H9	119.9
O3—S1—O1	113.85 (19)	N2—C10—C9	120.9 (3)
O2—S1—O4	107.39 (17)	N2—C10—C14	118.2 (2)
O3—S1—O4	98.78 (18)	C9—C10—C14	121.0 (3)
O1—S1—O4	105.86 (13)	N2—C11—C7	121.5 (2)
S1—O1—Cd	146.99 (14)	N2—C11—C12	118.8 (2)
O4^i^—O4—S1	108.4 (2)	C7—C11—C12	119.6 (2)
C1—N1—C12	118.9 (2)	N1—C12—C4	122.2 (3)
C1—N1—Cd	129.28 (18)	N1—C12—C11	119.2 (2)
C12—N1—Cd	109.09 (17)	C4—C12—C11	118.5 (2)
C10—N2—C11	119.8 (2)	C1—C13—H13A	109.5
C10—N2—Cd	125.77 (17)	C1—C13—H13B	109.5
C11—N2—Cd	112.87 (16)	H13A—C13—H13B	109.5
N1—C1—C2	121.3 (3)	C1—C13—H13C	109.5
N1—C1—C13	118.7 (3)	H13A—C13—H13C	109.5
C2—C1—C13	119.9 (3)	H13B—C13—H13C	109.5
C3—C2—C1	120.4 (3)	C10—C14—H14A	109.5
C3—C2—H2	119.8	C10—C14—H14B	109.5
C1—C2—H2	119.8	H14A—C14—H14B	109.5
C2—C3—C4	119.4 (3)	C10—C14—H14C	109.5
C2—C3—H3	120.3	H14A—C14—H14C	109.5
C4—C3—H3	120.3	H14B—C14—H14C	109.5
C3—C4—C12	117.5 (3)		
			
O2—S1—O1—Cd	135.8 (3)	C11—N2—C10—C14	−178.0 (2)
O3—S1—O1—Cd	−88.7 (3)	Cd—N2—C10—C14	17.2 (4)
O4—S1—O1—Cd	18.7 (3)	C8—C9—C10—N2	1.4 (5)
O2—S1—O4—O4^i^	−57.55 (19)	C8—C9—C10—C14	−178.5 (3)
O3—S1—O4—O4^i^	−179.04 (19)	C10—N2—C11—C7	−4.8 (4)
O1—S1—O4—O4^i^	62.98 (17)	Cd—N2—C11—C7	161.95 (19)
C12—N1—C1—C2	−5.1 (4)	C10—N2—C11—C12	173.3 (2)
Cd—N1—C1—C2	154.1 (2)	Cd—N2—C11—C12	−19.9 (3)
C12—N1—C1—C13	173.6 (3)	C8—C7—C11—N2	3.7 (4)
Cd—N1—C1—C13	−27.3 (4)	C6—C7—C11—N2	−177.9 (3)
N1—C1—C2—C3	2.4 (5)	C8—C7—C11—C12	−174.4 (2)
C13—C1—C2—C3	−176.2 (3)	C6—C7—C11—C12	4.0 (4)
C1—C2—C3—C4	2.0 (5)	C1—N1—C12—C4	3.4 (4)
C2—C3—C4—C12	−3.5 (5)	Cd—N1—C12—C4	−159.6 (2)
C2—C3—C4—C5	174.9 (3)	C1—N1—C12—C11	−173.7 (2)
C3—C4—C5—C6	−176.5 (3)	Cd—N1—C12—C11	23.2 (3)
C12—C4—C5—C6	1.9 (5)	C3—C4—C12—N1	0.9 (4)
C4—C5—C6—C7	−0.4 (5)	C5—C4—C12—N1	−177.6 (3)
C5—C6—C7—C8	175.8 (3)	C3—C4—C12—C11	178.0 (3)
C5—C6—C7—C11	−2.5 (5)	C5—C4—C12—C11	−0.4 (4)
C11—C7—C8—C9	−0.1 (5)	N2—C11—C12—N1	−3.4 (3)
C6—C7—C8—C9	−178.4 (3)	C7—C11—C12—N1	174.7 (2)
C7—C8—C9—C10	−2.4 (5)	N2—C11—C12—C4	179.4 (2)
C11—N2—C10—C9	2.2 (4)	C7—C11—C12—C4	−2.5 (4)
Cd—N2—C10—C9	−162.7 (2)		

Symmetry code: (i) −*x*, *y*, −*z*+1/2.

### (VI) Bis(3,4,7,8-tetramethy-1,10-phenanthroline-κ2N,N')(peroxodisulfato-κ2O,O')zinc(II) Crystal data

**Table:**

[Zn(S_2_O_8_)(C_16_H_16_N_2_)_2_]	*D*_x_ = 1.590 Mg m^−^^3^
*M**_r_* = 730.10	Mo *K*α radiation, λ = 0.71073 Å
Orthorhombic, *P**b**c**n*	Cell parameters from 3997 reflections
*a* = 15.6244 (2) Å	θ = 4.0–28.1°
*b* = 10.8803 (2) Å	µ = 1.00 mm^−^^1^
*c* = 17.9446 (3) Å	*T* = 294 K
*V* = 3050.55 (9) Å^3^	Blocks, colorless
*Z* = 4	0.35 × 0.20 × 0.16 mm
*F*(000) = 1512	

### (VI) Bis(3,4,7,8-tetramethy-1,10-phenanthroline-κ2N,N')(peroxodisulfato-κ2O,O')zinc(II) Data collection

**Table:**

Oxford Diffraction Gemini CCD S Ultra diffractometer	3341 reflections with *I* > 2σ(*I*)
ω scans, thick slices	*R*_int_ = 0.049
Absorption correction: multi-scan (CrysAlis PRO; Oxford Diffraction, 2009)	θ_max_ = 29.3°, θ_min_ = 3.8°
*T*_min_ = 0.76, *T*_max_ = 0.84	*h* = −21→21
63344 measured reflections	*k* = −14→14
3988 independent reflections	*l* = −23→23

### (VI) Bis(3,4,7,8-tetramethy-1,10-phenanthroline-κ2N,N')(peroxodisulfato-κ2O,O')zinc(II) Refinement

**Table:**

Refinement on *F*^2^	0 restraints
Least-squares matrix: full	Hydrogen site location: inferred from neighbouring sites
*R*[*F*^2^ > 2σ(*F*^2^)] = 0.033	H-atom parameters constrained
*wR*(*F*^2^) = 0.092	*w* = 1/[σ^2^(*F*_o_^2^) + (0.0436*P*)^2^ + 2.8456*P*] where *P* = (*F*_o_^2^ + 2*F*_c_^2^)/3
*S* = 1.04	(Δ/σ)_max_ < 0.001
3988 reflections	Δρ_max_ = 0.47 e Å^−^^3^
217 parameters	Δρ_min_ = −0.53 e Å^−^^3^

### (VI) Bis(3,4,7,8-tetramethy-1,10-phenanthroline-κ2N,N')(peroxodisulfato-κ2O,O')zinc(II) Special details

**Table:**

Geometry. All esds (except the esd in the dihedral angle between two l.s. planes) are estimated using the full covariance matrix. The cell esds are taken into account individually in the estimation of esds in distances, angles and torsion angles; correlations between esds in cell parameters are only used when they are defined by crystal symmetry. An approximate (isotropic) treatment of cell esds is used for estimating esds involving l.s. planes.

### (VI) Bis(3,4,7,8-tetramethy-1,10-phenanthroline-κ2N,N')(peroxodisulfato-κ2O,O')zinc(II) Fractional atomic coordinates and isotropic or equivalent isotropic displacement parameters (Å^2^)

**Table:**

	*x*	*y*	*z*	*U*_iso_*/*U*_eq_	
Zn1	0.0000	0.15234 (2)	0.2500	0.01734 (9)	
S1	−0.07734 (3)	0.42233 (4)	0.17271 (2)	0.02274 (11)	
O1	−0.05400 (9)	0.29339 (12)	0.18073 (7)	0.0249 (3)	
O2	−0.15306 (9)	0.45546 (14)	0.21252 (9)	0.0374 (4)	
O3	−0.06987 (11)	0.46565 (14)	0.09785 (8)	0.0389 (4)	
O4	0.00284 (9)	0.50125 (14)	0.20933 (8)	0.0313 (3)	
N1	0.11590 (9)	0.13279 (13)	0.19251 (8)	0.0190 (3)	
N2	−0.03181 (10)	0.01731 (13)	0.16421 (8)	0.0191 (3)	
C1	0.18924 (11)	0.18781 (16)	0.20877 (10)	0.0217 (3)	
H1	0.1887	0.2477	0.2458	0.026*	
C2	0.26765 (11)	0.16206 (16)	0.17409 (10)	0.0211 (4)	
C3	0.26914 (11)	0.07603 (16)	0.11707 (10)	0.0223 (4)	
C4	0.19057 (11)	0.01752 (16)	0.09742 (10)	0.0210 (3)	
C5	0.18323 (13)	−0.07255 (17)	0.03903 (10)	0.0262 (4)	
H5	0.2314	−0.0919	0.0109	0.031*	
C6	0.10858 (13)	−0.12971 (17)	0.02393 (10)	0.0253 (4)	
H6	0.1067	−0.1873	−0.0143	0.030*	
C7	0.03198 (12)	−0.10420 (16)	0.06522 (10)	0.0208 (3)	
C8	−0.04694 (13)	−0.16591 (16)	0.05399 (10)	0.0226 (4)	
C9	−0.11588 (12)	−0.13397 (17)	0.09853 (10)	0.0242 (4)	
C10	−0.10495 (12)	−0.04113 (16)	0.15199 (10)	0.0227 (4)	
H10	−0.1521	−0.0192	0.1807	0.027*	
C11	0.03636 (11)	−0.01425 (15)	0.12169 (9)	0.0186 (3)	
C12	0.11614 (11)	0.04729 (15)	0.13729 (9)	0.0182 (3)	
C13	0.34554 (12)	0.22736 (18)	0.20309 (11)	0.0265 (4)	
H13A	0.3835	0.2454	0.1624	0.040*	
H13B	0.3744	0.1758	0.2385	0.040*	
H13C	0.3286	0.3026	0.2268	0.040*	
C14	0.35083 (13)	0.0417 (2)	0.07834 (12)	0.0320 (4)	
H14A	0.3946	0.1000	0.0909	0.048*	
H14B	0.3419	0.0420	0.0254	0.048*	
H14C	0.3683	−0.0389	0.0940	0.048*	
C15	−0.05618 (14)	−0.26544 (18)	−0.00342 (11)	0.0301 (4)	
H15A	−0.1114	−0.2593	−0.0266	0.045*	
H15B	−0.0507	−0.3443	0.0202	0.045*	
H15C	−0.0123	−0.2564	−0.0405	0.045*	
C16	−0.20162 (13)	−0.1962 (2)	0.09203 (12)	0.0350 (5)	
H16A	−0.2241	−0.1837	0.0428	0.052*	
H16B	−0.2403	−0.1620	0.1280	0.052*	
H16C	−0.1951	−0.2827	0.1011	0.052*	

### (VI) Bis(3,4,7,8-tetramethy-1,10-phenanthroline-κ2N,N')(peroxodisulfato-κ2O,O')zinc(II) Atomic displacement parameters (Å^2^)

**Table:**

	*U*^11^	*U*^22^	*U*^33^	*U*^12^	*U*^13^	*U*^23^
Zn1	0.01923 (15)	0.01694 (15)	0.01584 (15)	0.000	0.00453 (10)	0.000
S1	0.0253 (2)	0.0201 (2)	0.0228 (2)	−0.00006 (16)	−0.00201 (17)	0.00241 (16)
O1	0.0321 (7)	0.0201 (6)	0.0225 (6)	0.0026 (5)	0.0000 (5)	0.0021 (5)
O2	0.0297 (8)	0.0318 (8)	0.0506 (10)	0.0035 (6)	0.0052 (7)	−0.0070 (7)
O3	0.0524 (10)	0.0348 (8)	0.0294 (8)	−0.0018 (7)	−0.0063 (7)	0.0121 (6)
O4	0.0350 (7)	0.0312 (7)	0.0276 (8)	−0.0083 (6)	−0.0022 (6)	0.0049 (6)
N1	0.0217 (7)	0.0183 (7)	0.0170 (7)	0.0003 (6)	0.0040 (6)	0.0000 (5)
N2	0.0221 (7)	0.0172 (7)	0.0179 (7)	0.0002 (6)	0.0029 (6)	0.0003 (5)
C1	0.0242 (9)	0.0183 (8)	0.0226 (9)	−0.0003 (7)	0.0050 (7)	−0.0015 (6)
C2	0.0211 (9)	0.0194 (8)	0.0228 (8)	0.0002 (6)	0.0039 (7)	0.0037 (6)
C3	0.0234 (9)	0.0207 (8)	0.0226 (8)	0.0042 (7)	0.0052 (7)	0.0037 (7)
C4	0.0238 (8)	0.0191 (8)	0.0200 (8)	0.0037 (7)	0.0050 (7)	0.0011 (6)
C5	0.0309 (10)	0.0252 (9)	0.0226 (9)	0.0058 (8)	0.0084 (7)	−0.0039 (7)
C6	0.0336 (10)	0.0221 (9)	0.0203 (9)	0.0046 (7)	0.0050 (7)	−0.0044 (7)
C7	0.0284 (9)	0.0169 (8)	0.0170 (8)	0.0028 (7)	0.0011 (7)	0.0014 (6)
C8	0.0322 (10)	0.0171 (8)	0.0185 (8)	0.0002 (7)	−0.0020 (7)	0.0006 (6)
C9	0.0287 (9)	0.0226 (9)	0.0212 (9)	−0.0028 (7)	−0.0014 (7)	0.0000 (7)
C10	0.0234 (8)	0.0227 (8)	0.0220 (8)	−0.0007 (7)	0.0030 (7)	0.0013 (7)
C11	0.0233 (8)	0.0163 (8)	0.0161 (8)	0.0021 (6)	0.0017 (6)	0.0015 (6)
C12	0.0236 (8)	0.0154 (7)	0.0156 (7)	0.0022 (6)	0.0031 (6)	0.0026 (6)
C13	0.0233 (9)	0.0252 (9)	0.0310 (10)	−0.0019 (7)	0.0045 (7)	0.0011 (7)
C14	0.0245 (9)	0.0356 (11)	0.0357 (11)	0.0023 (8)	0.0088 (8)	−0.0079 (9)
C15	0.0402 (11)	0.0245 (9)	0.0257 (9)	−0.0019 (8)	−0.0026 (8)	−0.0061 (7)
C16	0.0317 (10)	0.0402 (12)	0.0330 (11)	−0.0102 (9)	0.0006 (9)	−0.0098 (9)

### (VI) Bis(3,4,7,8-tetramethy-1,10-phenanthroline-κ2N,N')(peroxodisulfato-κ2O,O')zinc(II) Geometric parameters (Å, º)

**Table:**

Zn1—N1	2.0950 (14)	C5—H5	0.9300
Zn1—N1^i^	2.0951 (14)	C6—C7	1.435 (3)
Zn1—O1^i^	2.1476 (13)	C6—H6	0.9300
Zn1—O1	2.1476 (13)	C7—C11	1.410 (2)
Zn1—N2^i^	2.1853 (14)	C7—C8	1.418 (3)
Zn1—N2	2.1853 (14)	C8—C9	1.385 (3)
S1—O2	1.4281 (15)	C8—C15	1.502 (2)
S1—O3	1.4285 (15)	C9—C10	1.404 (3)
S1—O1	1.4567 (13)	C9—C16	1.506 (3)
S1—O4	1.6548 (15)	C10—H10	0.9300
O4—O4^i^	1.462 (3)	C11—C12	1.442 (2)
N1—C1	1.325 (2)	C13—H13A	0.9600
N1—C12	1.359 (2)	C13—H13B	0.9600
N2—C10	1.326 (2)	C13—H13C	0.9600
N2—C11	1.354 (2)	C14—H14A	0.9600
C1—C2	1.402 (2)	C14—H14B	0.9600
C1—H1	0.9300	C14—H14C	0.9600
C2—C3	1.387 (3)	C15—H15A	0.9600
C2—C13	1.502 (3)	C15—H15B	0.9600
C3—C4	1.427 (3)	C15—H15C	0.9600
C3—C14	1.500 (2)	C16—H16A	0.9600
C4—C12	1.403 (2)	C16—H16B	0.9600
C4—C5	1.439 (2)	C16—H16C	0.9600
C5—C6	1.349 (3)		
			
N1—Zn1—N1^i^	168.35 (8)	C5—C6—H6	119.1
N1—Zn1—O1^i^	91.03 (5)	C7—C6—H6	119.1
N1^i^—Zn1—O1^i^	97.30 (5)	C11—C7—C8	118.20 (16)
N1—Zn1—O1	97.30 (5)	C11—C7—C6	117.70 (17)
N1^i^—Zn1—O1	91.03 (5)	C8—C7—C6	124.08 (16)
N1^i^—Zn1—N2^i^	77.38 (6)	C9—C8—C7	118.38 (16)
O1—Zn1—O1^i^	88.78 (7)	C9—C8—C15	120.13 (17)
N1—Zn1—N2^i^	94.71 (6)	C7—C8—C15	121.49 (17)
O1^i^—Zn1—N2^i^	89.04 (5)	C8—C9—C10	118.70 (17)
O1—Zn1—N2^i^	167.83 (5)	C8—C9—C16	122.29 (17)
N1—Zn1—N2	77.37 (6)	C10—C9—C16	119.01 (17)
N1^i^—Zn1—N2	94.71 (6)	N2—C10—C9	124.26 (17)
O1^i^—Zn1—N2	167.83 (5)	N2—C10—H10	117.9
O1—Zn1—N2	89.04 (5)	C9—C10—H10	117.9
N2^i^—Zn1—N2	95.52 (8)	N2—C11—C7	122.86 (16)
O2—S1—O3	117.05 (10)	N2—C11—C12	116.90 (15)
O2—S1—O1	113.65 (9)	C7—C11—C12	120.23 (16)
O3—S1—O1	112.97 (9)	N1—C12—C4	122.13 (16)
O2—S1—O4	107.31 (9)	N1—C12—C11	117.19 (15)
O3—S1—O4	98.06 (9)	C4—C12—C11	120.67 (15)
O1—S1—O4	105.72 (8)	C2—C13—H13A	109.5
S1—O1—Zn1	147.54 (8)	C2—C13—H13B	109.5
O4^i^—O4—S1	110.51 (12)	H13A—C13—H13B	109.5
C1—N1—C12	117.87 (15)	C2—C13—H13C	109.5
C1—N1—Zn1	126.37 (12)	H13A—C13—H13C	109.5
C12—N1—Zn1	115.52 (11)	H13B—C13—H13C	109.5
C10—N2—C11	117.58 (15)	C3—C14—H14A	109.5
C10—N2—Zn1	129.41 (12)	C3—C14—H14B	109.5
C11—N2—Zn1	112.86 (11)	H14A—C14—H14B	109.5
N1—C1—C2	124.57 (16)	C3—C14—H14C	109.5
N1—C1—H1	117.7	H14A—C14—H14C	109.5
C2—C1—H1	117.7	H14B—C14—H14C	109.5
C3—C2—C1	118.48 (17)	C8—C15—H15A	109.5
C3—C2—C13	124.13 (16)	C8—C15—H15B	109.5
C1—C2—C13	117.36 (16)	H15A—C15—H15B	109.5
C2—C3—C4	117.96 (16)	C8—C15—H15C	109.5
C2—C3—C14	121.60 (17)	H15A—C15—H15C	109.5
C4—C3—C14	120.41 (16)	H15B—C15—H15C	109.5
C12—C4—C3	118.95 (16)	C9—C16—H16A	109.5
C12—C4—C5	117.53 (16)	C9—C16—H16B	109.5
C3—C4—C5	123.52 (16)	H16A—C16—H16B	109.5
C6—C5—C4	121.94 (17)	C9—C16—H16C	109.5
C6—C5—H5	119.0	H16A—C16—H16C	109.5
C4—C5—H5	119.0	H16B—C16—H16C	109.5
C5—C6—C7	121.88 (17)		
			
O2—S1—O1—Zn1	−75.62 (18)	C15—C8—C9—C10	179.58 (17)
O3—S1—O1—Zn1	147.93 (15)	C7—C8—C9—C16	−178.56 (18)
O4—S1—O1—Zn1	41.81 (18)	C15—C8—C9—C16	0.2 (3)
O2—S1—O4—O4^i^	43.02 (11)	C11—N2—C10—C9	0.6 (3)
O3—S1—O4—O4^i^	164.71 (10)	Zn1—N2—C10—C9	−174.53 (13)
O1—S1—O4—O4^i^	−78.59 (10)	C8—C9—C10—N2	−1.5 (3)
C12—N1—C1—C2	−1.5 (3)	C16—C9—C10—N2	177.88 (18)
Zn1—N1—C1—C2	172.58 (13)	C10—N2—C11—C7	0.9 (2)
N1—C1—C2—C3	2.1 (3)	Zn1—N2—C11—C7	176.86 (13)
N1—C1—C2—C13	−176.01 (17)	C10—N2—C11—C12	−178.04 (15)
C1—C2—C3—C4	−0.6 (3)	Zn1—N2—C11—C12	−2.09 (18)
C13—C2—C3—C4	177.41 (16)	C8—C7—C11—N2	−1.5 (3)
C1—C2—C3—C14	−178.75 (17)	C6—C7—C11—N2	−179.96 (16)
C13—C2—C3—C14	−0.7 (3)	C8—C7—C11—C12	177.39 (15)
C2—C3—C4—C12	−1.3 (2)	C6—C7—C11—C12	−1.0 (2)
C14—C3—C4—C12	176.83 (17)	C1—N1—C12—C4	−0.7 (2)
C2—C3—C4—C5	179.53 (17)	Zn1—N1—C12—C4	−175.37 (13)
C14—C3—C4—C5	−2.3 (3)	C1—N1—C12—C11	178.29 (15)
C12—C4—C5—C6	−1.9 (3)	Zn1—N1—C12—C11	3.58 (19)
C3—C4—C5—C6	177.26 (18)	C3—C4—C12—N1	2.1 (3)
C4—C5—C6—C7	0.0 (3)	C5—C4—C12—N1	−178.75 (16)
C5—C6—C7—C11	1.5 (3)	C3—C4—C12—C11	−176.87 (15)
C5—C6—C7—C8	−176.81 (18)	C5—C4—C12—C11	2.3 (2)
C11—C7—C8—C9	0.6 (2)	N2—C11—C12—N1	−0.9 (2)
C6—C7—C8—C9	178.92 (17)	C7—C11—C12—N1	−179.87 (15)
C11—C7—C8—C15	−178.16 (16)	N2—C11—C12—C4	178.08 (15)
C6—C7—C8—C15	0.2 (3)	C7—C11—C12—C4	−0.9 (2)
C7—C8—C9—C10	0.8 (3)		

Symmetry code: (i) −*x*, *y*, −*z*+1/2.

### (VII) Bis(3,4,7,8-tetramethy-1,10-phenanthroline-κ2N,N')(peroxodisulfato-κ2O,O')cadmium(II) Crystal data

**Table:**

[Cd(S_2_O_8_)(C_16_H_16_N_2_)_2_]	*Z* = 2
*M**_r_* = 777.13	*F*(000) = 792
Triclinic, *P*1	*D*_x_ = 1.658 Mg m^−^^3^
*a* = 8.601 (3) Å	Mo *K*α radiation, λ = 0.71069 Å
*b* = 11.063 (4) Å	Cell parameters from 6894 reflections
*c* = 16.932 (5) Å	θ = 3.8–27.7°
α = 98.788 (5)°	µ = 0.90 mm^−^^1^
β = 97.713 (5)°	*T* = 294 K
γ = 97.943 (5)°	Blocks, colorless
*V* = 1557.0 (9) Å^3^	0.28 × 0.16 × 0.14 mm

### (VII) Bis(3,4,7,8-tetramethy-1,10-phenanthroline-κ2N,N')(peroxodisulfato-κ2O,O')cadmium(II) Data collection

**Table:**

Oxford Diffraction Gemini CCD S Ultra diffractometer	6692 reflections with *I* > 2σ(*I*)
ω scans, thick slices	*R*_int_ = 0.057
Absorption correction: multi-scan (CrysAlis PRO; Oxford Diffraction, 2009)	θ_max_ = 29.6°, θ_min_ = 3.7°
*T*_min_ = 0.76, *T*_max_ = 0.84	*h* = −11→11
41025 measured reflections	*k* = −15→14
7888 independent reflections	*l* = −22→23

### (VII) Bis(3,4,7,8-tetramethy-1,10-phenanthroline-κ2N,N')(peroxodisulfato-κ2O,O')cadmium(II) Refinement

**Table:**

Refinement on *F*^2^	0 restraints
Least-squares matrix: full	Hydrogen site location: inferred from neighbouring sites
*R*[*F*^2^ > 2σ(*F*^2^)] = 0.033	H-atom parameters constrained
*wR*(*F*^2^) = 0.071	*w* = 1/[σ^2^(*F*_o_^2^) + (0.0258*P*)^2^ + 0.357*P*] where *P* = (*F*_o_^2^ + 2*F*_c_^2^)/3
*S* = 1.07	(Δ/σ)_max_ = 0.001
7888 reflections	Δρ_max_ = 0.54 e Å^−^^3^
432 parameters	Δρ_min_ = −0.57 e Å^−^^3^

### (VII) Bis(3,4,7,8-tetramethy-1,10-phenanthroline-κ2N,N')(peroxodisulfato-κ2O,O')cadmium(II) Special details

**Table:**

Geometry. All esds (except the esd in the dihedral angle between two l.s. planes) are estimated using the full covariance matrix. The cell esds are taken into account individually in the estimation of esds in distances, angles and torsion angles; correlations between esds in cell parameters are only used when they are defined by crystal symmetry. An approximate (isotropic) treatment of cell esds is used for estimating esds involving l.s. planes.

### (VII) Bis(3,4,7,8-tetramethy-1,10-phenanthroline-κ2N,N')(peroxodisulfato-κ2O,O')cadmium(II) Fractional atomic coordinates and isotropic or equivalent isotropic displacement parameters (Å^2^)

**Table:**

	*x*	*y*	*z*	*U*_iso_*/*U*_eq_	
Cd1	0.47097 (2)	0.33533 (2)	0.72920 (2)	0.01922 (6)	
S1	0.05625 (7)	0.35870 (6)	0.71408 (3)	0.02590 (13)	
S2	0.20697 (7)	0.06721 (6)	0.75623 (4)	0.02696 (13)	
O1	0.21331 (18)	0.37290 (16)	0.69045 (10)	0.0286 (4)	
O2	0.0350 (2)	0.45715 (18)	0.77528 (10)	0.0423 (5)	
O3	−0.06850 (19)	0.31860 (17)	0.64709 (10)	0.0334 (4)	
O4	0.0523 (2)	0.24624 (17)	0.76875 (10)	0.0345 (4)	
O5	0.06436 (19)	0.13190 (16)	0.71331 (10)	0.0338 (4)	
O6	0.1605 (2)	0.03554 (17)	0.82923 (10)	0.0362 (4)	
O7	0.2034 (2)	−0.03378 (16)	0.69173 (11)	0.0402 (4)	
O8	0.34975 (18)	0.15938 (15)	0.77001 (11)	0.0322 (4)	
N1	0.4486 (2)	0.23623 (17)	0.59702 (11)	0.0217 (4)	
N2	0.6357 (2)	0.45495 (17)	0.66216 (11)	0.0202 (4)	
C1	0.3553 (3)	0.1293 (2)	0.56479 (14)	0.0260 (5)	
H1	0.3023	0.0868	0.5992	0.031*	
C2	0.3309 (3)	0.0759 (2)	0.48279 (15)	0.0287 (5)	
C3	0.4100 (3)	0.1376 (2)	0.43121 (14)	0.0292 (5)	
C4	0.5109 (3)	0.2525 (2)	0.46361 (13)	0.0261 (5)	
C5	0.5991 (3)	0.3243 (3)	0.41633 (14)	0.0319 (6)	
H5	0.5911	0.2950	0.3612	0.038*	
C6	0.6933 (3)	0.4328 (3)	0.44856 (15)	0.0316 (6)	
H6	0.7466	0.4770	0.4150	0.038*	
C7	0.7140 (3)	0.4823 (2)	0.53374 (14)	0.0244 (5)	
C8	0.8184 (3)	0.5928 (2)	0.57091 (15)	0.0280 (5)	
C9	0.8314 (3)	0.6286 (2)	0.65367 (16)	0.0297 (6)	
C10	0.7359 (3)	0.5573 (2)	0.69553 (14)	0.0255 (5)	
H10	0.7432	0.5837	0.7509	0.031*	
C11	0.6269 (2)	0.4147 (2)	0.58217 (13)	0.0201 (5)	
C12	0.5256 (2)	0.2986 (2)	0.54680 (13)	0.0205 (5)	
C13	0.2189 (3)	−0.0455 (3)	0.45618 (17)	0.0414 (7)	
H13A	0.2722	−0.1055	0.4282	0.062*	
H13B	0.1276	−0.0336	0.4207	0.062*	
H13C	0.1856	−0.0747	0.5028	0.062*	
C14	0.3930 (4)	0.0845 (3)	0.34201 (16)	0.0467 (7)	
H14A	0.3078	0.0153	0.3282	0.070*	
H14B	0.4903	0.0575	0.3307	0.070*	
H14C	0.3700	0.1470	0.3106	0.070*	
C15	0.9139 (3)	0.6671 (3)	0.52125 (17)	0.0409 (7)	
H15A	0.9716	0.7417	0.5551	0.061*	
H15B	0.8436	0.6879	0.4782	0.061*	
H15C	0.9874	0.6190	0.4988	0.061*	
C16	0.9451 (3)	0.7398 (3)	0.70052 (18)	0.0469 (8)	
H16A	0.9266	0.8118	0.6777	0.070*	
H16B	1.0523	0.7264	0.6978	0.070*	
H16C	0.9289	0.7522	0.7560	0.070*	
N21	0.4943 (2)	0.51270 (17)	0.82643 (10)	0.0198 (4)	
N22	0.6494 (2)	0.31779 (18)	0.84169 (11)	0.0219 (4)	
C21	0.4298 (3)	0.6120 (2)	0.81585 (14)	0.0242 (5)	
H21	0.3598	0.6073	0.7682	0.029*	
C22	0.4590 (3)	0.7236 (2)	0.87092 (14)	0.0253 (5)	
C23	0.5539 (3)	0.7279 (2)	0.94438 (14)	0.0252 (5)	
C24	0.6237 (2)	0.6230 (2)	0.95815 (13)	0.0230 (5)	
C25	0.7238 (3)	0.6180 (2)	1.03197 (14)	0.0283 (5)	
H25	0.7414	0.6853	1.0742	0.034*	
C26	0.7930 (3)	0.5181 (2)	1.04171 (14)	0.0290 (6)	
H26	0.8567	0.5182	1.0907	0.035*	
C27	0.7714 (3)	0.4113 (2)	0.97874 (14)	0.0254 (5)	
C28	0.8425 (3)	0.3054 (3)	0.98739 (15)	0.0289 (6)	
C29	0.8136 (3)	0.2077 (2)	0.92287 (16)	0.0302 (6)	
C30	0.7184 (3)	0.2201 (2)	0.85173 (15)	0.0282 (5)	
H30	0.7025	0.1550	0.8082	0.034*	
C31	0.6738 (2)	0.4133 (2)	0.90545 (13)	0.0204 (5)	
C32	0.5953 (2)	0.5188 (2)	0.89612 (12)	0.0192 (5)	
C33	0.3872 (3)	0.8322 (2)	0.84782 (17)	0.0366 (6)	
H33A	0.3236	0.8597	0.8873	0.055*	
H33B	0.3216	0.8077	0.7957	0.055*	
H33C	0.4704	0.8985	0.8457	0.055*	
C34	0.5838 (3)	0.8423 (2)	1.00842 (16)	0.0383 (6)	
H34A	0.5273	0.9039	0.9894	0.058*	
H34B	0.6956	0.8740	1.0200	0.058*	
H34C	0.5476	0.8220	1.0567	0.058*	
C35	0.9459 (3)	0.3000 (3)	1.06551 (16)	0.0418 (7)	
H35A	0.9709	0.2179	1.0644	0.063*	
H35B	0.8903	0.3204	1.1100	0.063*	
H35C	1.0423	0.3582	1.0719	0.063*	
C36	0.8790 (3)	0.0884 (3)	0.92540 (19)	0.0466 (7)	
H36A	0.8525	0.0550	0.9719	0.070*	
H36B	0.9925	0.1045	0.9288	0.070*	
H36C	0.8337	0.0299	0.8772	0.070*	

### (VII) Bis(3,4,7,8-tetramethy-1,10-phenanthroline-κ2N,N')(peroxodisulfato-κ2O,O')cadmium(II) Atomic displacement parameters (Å^2^)

**Table:**

	*U*^11^	*U*^22^	*U*^33^	*U*^12^	*U*^13^	*U*^23^
Cd1	0.02200 (9)	0.01855 (9)	0.01524 (9)	0.00058 (6)	0.00083 (6)	0.00144 (6)
S1	0.0251 (3)	0.0305 (3)	0.0203 (3)	0.0087 (2)	−0.0032 (2)	0.0011 (2)
S2	0.0275 (3)	0.0217 (3)	0.0298 (3)	−0.0027 (2)	0.0021 (2)	0.0066 (3)
O1	0.0237 (8)	0.0303 (10)	0.0302 (9)	0.0031 (7)	−0.0029 (7)	0.0073 (7)
O2	0.0526 (12)	0.0470 (12)	0.0256 (10)	0.0267 (10)	−0.0045 (8)	−0.0061 (8)
O3	0.0268 (9)	0.0404 (11)	0.0285 (9)	0.0075 (8)	−0.0071 (7)	0.0006 (8)
O4	0.0375 (10)	0.0416 (12)	0.0276 (9)	0.0128 (8)	0.0068 (8)	0.0085 (8)
O5	0.0339 (9)	0.0285 (10)	0.0333 (10)	−0.0021 (8)	−0.0063 (8)	0.0036 (8)
O6	0.0416 (10)	0.0378 (11)	0.0325 (10)	0.0050 (8)	0.0091 (8)	0.0145 (8)
O7	0.0562 (12)	0.0229 (10)	0.0384 (11)	−0.0037 (8)	0.0110 (9)	0.0014 (8)
O8	0.0249 (8)	0.0239 (9)	0.0449 (11)	−0.0048 (7)	−0.0021 (8)	0.0118 (8)
N1	0.0247 (10)	0.0216 (10)	0.0182 (9)	0.0033 (8)	0.0032 (8)	0.0023 (8)
N2	0.0184 (9)	0.0222 (10)	0.0197 (9)	0.0035 (7)	0.0004 (7)	0.0051 (8)
C1	0.0308 (13)	0.0234 (13)	0.0219 (12)	0.0020 (10)	0.0030 (10)	0.0009 (10)
C2	0.0312 (13)	0.0239 (13)	0.0273 (13)	0.0086 (10)	−0.0034 (10)	−0.0033 (10)
C3	0.0337 (13)	0.0335 (15)	0.0197 (12)	0.0161 (11)	−0.0003 (10)	−0.0031 (10)
C4	0.0292 (12)	0.0339 (14)	0.0179 (11)	0.0151 (10)	0.0032 (9)	0.0046 (10)
C5	0.0388 (14)	0.0442 (17)	0.0176 (12)	0.0176 (12)	0.0087 (10)	0.0073 (11)
C6	0.0338 (14)	0.0438 (17)	0.0261 (13)	0.0148 (12)	0.0120 (11)	0.0192 (12)
C7	0.0197 (11)	0.0321 (14)	0.0264 (12)	0.0101 (10)	0.0055 (9)	0.0138 (10)
C8	0.0181 (11)	0.0359 (15)	0.0363 (14)	0.0088 (10)	0.0053 (10)	0.0208 (12)
C9	0.0207 (12)	0.0295 (14)	0.0380 (15)	−0.0008 (10)	−0.0004 (10)	0.0117 (11)
C10	0.0192 (11)	0.0324 (14)	0.0223 (12)	−0.0003 (10)	−0.0022 (9)	0.0055 (10)
C11	0.0179 (10)	0.0264 (13)	0.0192 (11)	0.0092 (9)	0.0029 (9)	0.0089 (9)
C12	0.0196 (11)	0.0244 (12)	0.0188 (11)	0.0090 (9)	0.0018 (9)	0.0038 (9)
C13	0.0487 (17)	0.0294 (15)	0.0359 (15)	−0.0007 (12)	−0.0062 (13)	−0.0086 (12)
C14	0.0608 (19)	0.052 (2)	0.0227 (14)	0.0156 (15)	0.0005 (13)	−0.0085 (13)
C15	0.0316 (14)	0.0490 (18)	0.0494 (17)	0.0053 (12)	0.0117 (13)	0.0283 (15)
C16	0.0359 (15)	0.0462 (19)	0.0509 (18)	−0.0166 (13)	−0.0026 (13)	0.0140 (15)
N21	0.0197 (9)	0.0229 (10)	0.0166 (9)	0.0025 (8)	0.0021 (7)	0.0040 (8)
N22	0.0216 (9)	0.0223 (10)	0.0221 (10)	0.0027 (8)	0.0038 (8)	0.0055 (8)
C21	0.0245 (12)	0.0265 (13)	0.0215 (12)	0.0044 (10)	0.0033 (9)	0.0039 (10)
C22	0.0254 (12)	0.0225 (13)	0.0276 (13)	−0.0005 (9)	0.0077 (10)	0.0043 (10)
C23	0.0263 (12)	0.0230 (13)	0.0240 (12)	−0.0041 (9)	0.0092 (10)	−0.0005 (10)
C24	0.0200 (11)	0.0293 (13)	0.0167 (11)	−0.0045 (9)	0.0045 (9)	0.0011 (9)
C25	0.0272 (12)	0.0361 (15)	0.0166 (11)	−0.0052 (11)	0.0020 (9)	−0.0004 (10)
C26	0.0238 (12)	0.0446 (16)	0.0151 (11)	−0.0036 (11)	−0.0018 (9)	0.0070 (11)
C27	0.0180 (11)	0.0386 (15)	0.0205 (12)	0.0006 (10)	0.0043 (9)	0.0102 (10)
C28	0.0171 (11)	0.0443 (16)	0.0290 (13)	0.0045 (10)	0.0065 (10)	0.0163 (12)
C29	0.0224 (12)	0.0385 (16)	0.0369 (14)	0.0115 (11)	0.0101 (11)	0.0185 (12)
C30	0.0266 (12)	0.0297 (14)	0.0301 (13)	0.0070 (10)	0.0067 (10)	0.0063 (11)
C31	0.0160 (10)	0.0253 (12)	0.0188 (11)	−0.0020 (9)	0.0039 (8)	0.0042 (9)
C32	0.0159 (10)	0.0246 (12)	0.0160 (11)	−0.0019 (9)	0.0039 (8)	0.0037 (9)
C33	0.0435 (15)	0.0251 (14)	0.0403 (16)	0.0055 (12)	0.0034 (12)	0.0056 (12)
C34	0.0467 (16)	0.0282 (15)	0.0338 (15)	−0.0008 (12)	0.0056 (12)	−0.0073 (11)
C35	0.0306 (14)	0.065 (2)	0.0366 (15)	0.0123 (13)	0.0039 (12)	0.0256 (15)
C36	0.0467 (17)	0.0494 (19)	0.0535 (19)	0.0232 (14)	0.0117 (14)	0.0223 (15)

### (VII) Bis(3,4,7,8-tetramethy-1,10-phenanthroline-κ2N,N')(peroxodisulfato-κ2O,O')cadmium(II) Geometric parameters (Å, º)

**Table:**

Cd1—N1	2.3075 (19)	C14—H14C	0.9600
Cd1—O8	2.3232 (18)	C15—H15A	0.9600
Cd1—N21	2.327 (2)	C15—H15B	0.9600
Cd1—N2	2.3278 (19)	C15—H15C	0.9600
Cd1—N22	2.3304 (19)	C16—H16A	0.9600
Cd1—O1	2.3371 (19)	C16—H16B	0.9600
S1—O3	1.4222 (17)	C16—H16C	0.9600
S1—O2	1.4333 (19)	N21—C21	1.322 (3)
S1—O1	1.4544 (18)	N21—C32	1.355 (3)
S1—O4	1.6597 (19)	N22—C30	1.325 (3)
S2—O6	1.4272 (18)	N22—C31	1.362 (3)
S2—O7	1.4320 (19)	C21—C22	1.399 (3)
S2—O8	1.4514 (17)	C21—H21	0.9300
S2—O5	1.6454 (18)	C22—C23	1.384 (3)
O4—O5	1.480 (2)	C22—C33	1.504 (3)
N1—C1	1.328 (3)	C23—C24	1.414 (3)
N1—C12	1.357 (3)	C23—C34	1.503 (3)
N2—C10	1.322 (3)	C24—C32	1.405 (3)
N2—C11	1.348 (3)	C24—C25	1.432 (3)
C1—C2	1.399 (3)	C25—C26	1.347 (4)
C1—H1	0.9300	C25—H25	0.9300
C2—C3	1.380 (3)	C26—C27	1.438 (3)
C2—C13	1.509 (4)	C26—H26	0.9300
C3—C4	1.423 (4)	C27—C31	1.407 (3)
C3—C14	1.515 (3)	C27—C28	1.412 (3)
C4—C12	1.406 (3)	C28—C29	1.384 (4)
C4—C5	1.428 (3)	C28—C35	1.506 (3)
C5—C6	1.344 (4)	C29—C30	1.399 (3)
C5—H5	0.9300	C29—C36	1.509 (4)
C6—C7	1.440 (3)	C30—H30	0.9300
C6—H6	0.9300	C31—C32	1.445 (3)
C7—C11	1.411 (3)	C33—H33A	0.9600
C7—C8	1.417 (4)	C33—H33B	0.9600
C8—C9	1.382 (4)	C33—H33C	0.9600
C8—C15	1.505 (3)	C34—H34A	0.9600
C9—C10	1.395 (3)	C34—H34B	0.9600
C9—C16	1.503 (4)	C34—H34C	0.9600
C10—H10	0.9300	C35—H35A	0.9600
C11—C12	1.443 (3)	C35—H35B	0.9600
C13—H13A	0.9600	C35—H35C	0.9600
C13—H13B	0.9600	C36—H36A	0.9600
C13—H13C	0.9600	C36—H36B	0.9600
C14—H14A	0.9600	C36—H36C	0.9600
C14—H14B	0.9600		
			
N1—Cd1—O8	92.85 (7)	C3—C14—H14C	109.5
N1—Cd1—N21	152.02 (7)	H14A—C14—H14C	109.5
O8—Cd1—N21	112.40 (7)	H14B—C14—H14C	109.5
N1—Cd1—N2	71.27 (7)	C8—C15—H15A	109.5
O8—Cd1—N2	158.54 (6)	C8—C15—H15B	109.5
N21—Cd1—N2	86.65 (7)	H15A—C15—H15B	109.5
N1—Cd1—N22	128.61 (7)	C8—C15—H15C	109.5
O8—Cd1—N22	77.30 (6)	H15A—C15—H15C	109.5
N21—Cd1—N22	71.29 (7)	H15B—C15—H15C	109.5
N2—Cd1—N22	101.00 (7)	C9—C16—H16A	109.5
N1—Cd1—O1	85.06 (6)	C9—C16—H16B	109.5
O8—Cd1—O1	85.92 (6)	H16A—C16—H16B	109.5
N21—Cd1—O1	84.94 (6)	C9—C16—H16C	109.5
N2—Cd1—O1	106.32 (7)	H16A—C16—H16C	109.5
N22—Cd1—O1	142.43 (6)	H16B—C16—H16C	109.5
O3—S1—O2	117.21 (11)	C21—N21—C32	117.7 (2)
O3—S1—O1	113.08 (11)	C21—N21—Cd1	125.21 (15)
O2—S1—O1	113.93 (12)	C32—N21—Cd1	116.69 (14)
O3—S1—O4	106.56 (11)	C30—N22—C31	117.3 (2)
O2—S1—O4	98.35 (11)	C30—N22—Cd1	126.18 (16)
O1—S1—O4	105.54 (9)	C31—N22—Cd1	116.25 (14)
O6—S2—O7	116.46 (11)	N21—C21—C22	125.0 (2)
O6—S2—O8	113.18 (11)	N21—C21—H21	117.5
O7—S2—O8	114.04 (11)	C22—C21—H21	117.5
O6—S2—O5	107.04 (10)	C23—C22—C21	117.6 (2)
O7—S2—O5	98.35 (11)	C23—C22—C33	123.4 (2)
O8—S2—O5	105.77 (10)	C21—C22—C33	119.0 (2)
S1—O1—Cd1	141.34 (10)	C22—C23—C24	118.9 (2)
O5—O4—S1	106.47 (12)	C22—C23—C34	120.8 (2)
O4—O5—S2	108.11 (12)	C24—C23—C34	120.4 (2)
S2—O8—Cd1	144.53 (10)	C32—C24—C23	118.6 (2)
C1—N1—C12	117.72 (19)	C32—C24—C25	118.2 (2)
C1—N1—Cd1	125.25 (15)	C23—C24—C25	123.2 (2)
C12—N1—Cd1	116.71 (15)	C26—C25—C24	121.5 (2)
C10—N2—C11	117.90 (19)	C26—C25—H25	119.2
C10—N2—Cd1	125.90 (15)	C24—C25—H25	119.2
C11—N2—Cd1	116.20 (15)	C25—C26—C27	122.0 (2)
N1—C1—C2	124.8 (2)	C25—C26—H26	119.0
N1—C1—H1	117.6	C27—C26—H26	119.0
C2—C1—H1	117.6	C31—C27—C28	119.0 (2)
C3—C2—C1	118.1 (2)	C31—C27—C26	117.9 (2)
C3—C2—C13	124.0 (2)	C28—C27—C26	123.2 (2)
C1—C2—C13	117.8 (2)	C29—C28—C27	118.4 (2)
C2—C3—C4	118.7 (2)	C29—C28—C35	121.5 (2)
C2—C3—C14	121.3 (2)	C27—C28—C35	120.2 (2)
C4—C3—C14	120.0 (2)	C28—C29—C30	118.3 (2)
C12—C4—C3	118.6 (2)	C28—C29—C36	123.4 (2)
C12—C4—C5	117.7 (2)	C30—C29—C36	118.3 (3)
C3—C4—C5	123.7 (2)	N22—C30—C29	124.9 (2)
C6—C5—C4	122.4 (2)	N22—C30—H30	117.5
C6—C5—H5	118.8	C29—C30—H30	117.5
C4—C5—H5	118.8	N22—C31—C27	122.1 (2)
C5—C6—C7	121.7 (2)	N22—C31—C32	117.88 (19)
C5—C6—H6	119.1	C27—C31—C32	120.0 (2)
C7—C6—H6	119.1	N21—C32—C24	122.0 (2)
C11—C7—C8	119.0 (2)	N21—C32—C31	117.77 (19)
C11—C7—C6	117.5 (2)	C24—C32—C31	120.3 (2)
C8—C7—C6	123.6 (2)	C22—C33—H33A	109.5
C9—C8—C7	118.0 (2)	C22—C33—H33B	109.5
C9—C8—C15	121.5 (2)	H33A—C33—H33B	109.5
C7—C8—C15	120.5 (2)	C22—C33—H33C	109.5
C8—C9—C10	118.4 (2)	H33A—C33—H33C	109.5
C8—C9—C16	122.9 (2)	H33B—C33—H33C	109.5
C10—C9—C16	118.7 (2)	C23—C34—H34A	109.5
N2—C10—C9	124.8 (2)	C23—C34—H34B	109.5
N2—C10—H10	117.6	H34A—C34—H34B	109.5
C9—C10—H10	117.6	C23—C34—H34C	109.5
N2—C11—C7	121.8 (2)	H34A—C34—H34C	109.5
N2—C11—C12	117.89 (19)	H34B—C34—H34C	109.5
C7—C11—C12	120.3 (2)	C28—C35—H35A	109.5
N1—C12—C4	122.1 (2)	C28—C35—H35B	109.5
N1—C12—C11	117.48 (19)	H35A—C35—H35B	109.5
C4—C12—C11	120.4 (2)	C28—C35—H35C	109.5
C2—C13—H13A	109.5	H35A—C35—H35C	109.5
C2—C13—H13B	109.5	H35B—C35—H35C	109.5
H13A—C13—H13B	109.5	C29—C36—H36A	109.5
C2—C13—H13C	109.5	C29—C36—H36B	109.5
H13A—C13—H13C	109.5	H36A—C36—H36B	109.5
H13B—C13—H13C	109.5	C29—C36—H36C	109.5
C3—C14—H14A	109.5	H36A—C36—H36C	109.5
C3—C14—H14B	109.5	H36B—C36—H36C	109.5
H14A—C14—H14B	109.5		
			
O3—S1—O1—Cd1	−139.00 (15)	C3—C4—C12—C11	179.9 (2)
O2—S1—O1—Cd1	83.87 (18)	C5—C4—C12—C11	0.0 (3)
O4—S1—O1—Cd1	−22.89 (19)	N2—C11—C12—N1	−1.5 (3)
O3—S1—O4—O5	55.31 (14)	C7—C11—C12—N1	178.08 (19)
O2—S1—O4—O5	177.03 (13)	N2—C11—C12—C4	179.56 (19)
O1—S1—O4—O5	−65.17 (14)	C7—C11—C12—C4	−0.9 (3)
S1—O4—O5—S2	129.36 (11)	C32—N21—C21—C22	0.6 (3)
O6—S2—O5—O4	62.22 (15)	Cd1—N21—C21—C22	−172.20 (16)
O7—S2—O5—O4	−176.72 (13)	N21—C21—C22—C23	−4.6 (3)
O8—S2—O5—O4	−58.73 (15)	N21—C21—C22—C33	175.2 (2)
O6—S2—O8—Cd1	−139.60 (18)	C21—C22—C23—C24	3.7 (3)
O7—S2—O8—Cd1	84.2 (2)	C33—C22—C23—C24	−176.1 (2)
O5—S2—O8—Cd1	−22.7 (2)	C21—C22—C23—C34	−176.8 (2)
C12—N1—C1—C2	0.2 (3)	C33—C22—C23—C34	3.4 (3)
Cd1—N1—C1—C2	173.51 (17)	C22—C23—C24—C32	0.8 (3)
N1—C1—C2—C3	0.6 (4)	C34—C23—C24—C32	−178.8 (2)
N1—C1—C2—C13	−179.2 (2)	C22—C23—C24—C25	180.0 (2)
C1—C2—C3—C4	−0.6 (3)	C34—C23—C24—C25	0.4 (3)
C13—C2—C3—C4	179.2 (2)	C32—C24—C25—C26	1.7 (3)
C1—C2—C3—C14	178.5 (2)	C23—C24—C25—C26	−177.4 (2)
C13—C2—C3—C14	−1.8 (4)	C24—C25—C26—C27	0.4 (3)
C2—C3—C4—C12	−0.2 (3)	C25—C26—C27—C31	−0.3 (3)
C14—C3—C4—C12	−179.3 (2)	C25—C26—C27—C28	−179.9 (2)
C2—C3—C4—C5	179.7 (2)	C31—C27—C28—C29	0.3 (3)
C14—C3—C4—C5	0.6 (4)	C26—C27—C28—C29	179.9 (2)
C12—C4—C5—C6	−0.2 (3)	C31—C27—C28—C35	−179.2 (2)
C3—C4—C5—C6	179.8 (2)	C26—C27—C28—C35	0.4 (3)
C4—C5—C6—C7	1.4 (4)	C27—C28—C29—C30	1.5 (3)
C5—C6—C7—C11	−2.2 (3)	C35—C28—C29—C30	−179.0 (2)
C5—C6—C7—C8	176.6 (2)	C27—C28—C29—C36	−178.4 (2)
C11—C7—C8—C9	0.7 (3)	C35—C28—C29—C36	1.2 (4)
C6—C7—C8—C9	−178.0 (2)	C31—N22—C30—C29	0.4 (3)
C11—C7—C8—C15	179.7 (2)	Cd1—N22—C30—C29	−173.78 (17)
C6—C7—C8—C15	1.0 (3)	C28—C29—C30—N22	−1.9 (4)
C7—C8—C9—C10	−2.9 (3)	C36—C29—C30—N22	177.9 (2)
C15—C8—C9—C10	178.1 (2)	C30—N22—C31—C27	1.6 (3)
C7—C8—C9—C16	176.4 (2)	Cd1—N22—C31—C27	176.34 (15)
C15—C8—C9—C16	−2.6 (4)	C30—N22—C31—C32	−178.18 (19)
C11—N2—C10—C9	1.4 (3)	Cd1—N22—C31—C32	−3.5 (2)
Cd1—N2—C10—C9	−178.82 (17)	C28—C27—C31—N22	−2.0 (3)
C8—C9—C10—N2	1.9 (4)	C26—C27—C31—N22	178.40 (19)
C16—C9—C10—N2	−177.3 (2)	C28—C27—C31—C32	177.83 (19)
C10—N2—C11—C7	−3.7 (3)	C26—C27—C31—C32	−1.8 (3)
Cd1—N2—C11—C7	176.52 (15)	C21—N21—C32—C24	4.3 (3)
C10—N2—C11—C12	175.92 (19)	Cd1—N21—C32—C24	177.72 (15)
Cd1—N2—C11—C12	−3.9 (2)	C21—N21—C32—C31	−176.02 (18)
C8—C7—C11—N2	2.7 (3)	Cd1—N21—C32—C31	−2.6 (2)
C6—C7—C11—N2	−178.5 (2)	C23—C24—C32—N21	−5.0 (3)
C8—C7—C11—C12	−176.92 (19)	C25—C24—C32—N21	175.79 (19)
C6—C7—C11—C12	1.9 (3)	C23—C24—C32—C31	175.35 (19)
C1—N1—C12—C4	−1.0 (3)	C25—C24—C32—C31	−3.9 (3)
Cd1—N1—C12—C4	−174.88 (16)	N22—C31—C32—N21	4.1 (3)
C1—N1—C12—C11	−179.93 (19)	C27—C31—C32—N21	−175.70 (18)
Cd1—N1—C12—C11	6.2 (2)	N22—C31—C32—C24	−176.24 (18)
C3—C4—C12—N1	1.0 (3)	C27—C31—C32—C24	4.0 (3)
C5—C4—C12—N1	−178.9 (2)		
